# Barriers and facilitators for the implementation of preventative mental health interventions among secondary schools in high-income countries: a systematic review

**DOI:** 10.1007/s00787-025-02796-5

**Published:** 2025-06-30

**Authors:** Sarah K. Schäfer, Sophie Streit, Christian G. Schäfer, Laila B. Roembell, Marie Corneli, Lea M. Schaubruch, Michèle Wessa, Klaus Lieb, Monika Equit, Daniela Fuhr

**Affiliations:** 1 Clinical Psychology and Psychotherapy for Children and Adolescents, Institute for Psychology, Technische Universität Braunschweig, Braunschweig, Germany; 2 Leibniz Institute for Resilience Research, Mainz, Germany; 3 Division of Clinical Psychology and Psychotherapy, Department of Psychology, Saarland University, Saarbrücken, Germany; 4 DKFZ Hector Cancer Institute at the University Medical Center Mannheim, Mannheim, Germany; 5 German Cancer Research Center (DKFZ) Heidelberg, Division Cancer Survivorship and Psychological Resilience, Heidelberg, Germany; 6 Central Institute of Mental Health, Department of Neuropsychology and Psychological Resilience Research, Mannheim, Germany; 7 Department of Psychiatry and Psychotherapy, University Medical Center of Johannes Gutenberg University Mainz, Mainz, Germany; 8 Leibniz Institute for Prevention Research and Epidemiology, Bremen, Germany; 9 University of Bremen, Health Sciences, Bremen, Germany; 10 London School of Hygiene and Tropical Medicine, Department of Health Services Research and Policy, London, United Kingdom

**Keywords:** Children, Adolescents, Pupils, Mental health, Implementation, Systematic review

## Abstract

Recent societal crises have negatively impacted the mental health of adolescents, emphasizing the need for effective preventative mental health interventions. Schools as key environments for young people offer opportunities to deliver those interventions at low threshold. However, many interventions are not sustained beyond initial funding periods due to persistent implementation barriers, contributing to a significant gap between intervention development and long-term practice. This systematic review synthesizes qualitative research on barriers and facilitators affecting the implementation of school-based interventions in secondary schools in high-income countries. Seven databases were searched for qualitative and mixed-methods studies on the implementation of preventative school-based mental health interventions in regular secondary schools. Implementation determinants were synthesized using the updated version of the Consolidated Framework for Implementation Research. Study quality was assessed using modified checklists of the Critical Appraisal Skills Programme. Twenty-six studies, involving 5 to 8,630 pupils, met the inclusion criteria. A total of 336 implementation factors were identified, with barriers (*N* = 207; 61.6%) outweighing facilitators (*N* = 129; 38.4%). Barriers included scheduling conflicts, low prioritization of mental health, and logistical challenges. Key facilitators were leadership support, age-appropriate intervention design, and staff engagement. Sustained success of preventative mental health interventions requires integrating programs into school structures, aligning with academic priorities, and addressing resource limitations. Future research should focus on adapting interventions to fit school contexts and strengthening leadership and community support. Understanding multidimensional, interacting implementation factors is essential for creating sustainable interventions that positively impact adolescent mental health.

**Supplementary Information:**

The online version contains supplementary material available at 10.1007/s00787-025-02796-5.

## Introduction

Within the last few years societies were exposed to a growing number of societal crises (e.g., the COVID-19 pandemic, climate crises) which resulted in heightened mental distress among the general population [1, 2]. Evidence showed that children and adolescents were proportionally more burdened by those crises than adults [3, 4], and this was especially the case during the COVID-19 pandemic [3, 5–7]. Preliminary evidence also points to more severe negative mental health consequences at younger ages in children who are exposed to the climate crises [8, 9] and the war in the Ukraine [10], even in non-exposed adolescents [11]. Increased mental distress and disorders may lead to severe negative consequences for children and their families—reduced quality of life, impaired social participation, and lower academic achievements [12, 13]. Moreover, mental disorders at younger ages are associated with worse mental and physical health in later life, resulting in potentially life-long major individual and societal costs [14, 15].

These costs resulted in a large number of calls to intensify efforts in promoting mental health and preventing mental disorders in children and adolescents within the last few years [16–18]. Many of those initiatives point to schools as potential settings for low-intensity health promotion and prevention measures [19–21]. As academic institutions, schools play a crucial role in the social, emotional and cognitive development of children and adolescents [22]. Given the substantial amount of time that young people spend in these settings, and the ability of public schools to reach out to diverse social groups of pupils at low threshold, regardless of their interests, social status or family circumstances [22, 23], schools are considered optimal environments to address mental health topics [24]. Although schools are increasingly recognized as potential venues of mental health promotion [25], education about mental health and mental disorders or maintaining mental health in the face of stress is not yet a regular part of the curriculum in most public schools, leaving relevant potential for mental health promotion and prevention untapped [26].

At the same time, a substantial number of research projects examined the effects of school-based psychosocial interventions preventing mental disorders and/or promoting mental health [27]. Systematic reviews focused on their efficacy and/or effectiveness demonstrated that these interventions had small favorable effects in reducing internalizing symptoms [24, 28, 29], and resulted in small improvements in mental health knowledge [23, 30]. However, they did not significantly influence mental health stigma, attitudes towards mental health, or help-seeking behavior [30]. Moreover, those reviews highlighted substantial heterogeneity in how the interventions were implemented [24, 28, 29].

However, many of those interventions examined in fully powered efficacy or effectiveness trials were not sustained beyond the period of funding [31] or considered to be implemented in the wider educational system [35]. Reasons for not continuing those interventions might be heterogeneous and include barriers of implementation at different levels [32]. Such barriers—and, conversely, potential facilitators—can result from the intervention itself (also called innovation), the proximal or distal setting of implementation, individuals involved in the implementation, and the process of implementation itself [33].

Despite broad consensus on the importance of rollout [34] and challenges reported for implementing interventions in school settings [35], barriers and facilitators of school-based psychosocial interventions are still poorly understood [24]. While many studies have focused primarily on evaluating the efficacy or effectiveness of interventions, implementation remains a critical yet underexplored component of the translational process in intervention research [36]. Limited understanding of implementation processes likely contributes to the persistent implementation gap, where numerous psychosocial interventions are developed and tested, but few are ultimately adopted in real-world settings [36]. Narrowing this gap could significantly advance both research and practice. Embedding implementation research within real-world school contexts can generate valuable theoretical insights into the relationship between implementation processes and intervention outcomes, providing crucial feedback to guide future research [34, 36]. From a practical perspective, anticipating and addressing implementation challenges during the design of new interventions and the refinement of existing programs is essential. Thus, expanding knowledge of barriers and facilitators could improve the accessibility and sustainability of prevention and mental health promotion where they are most needed.

Existing evidence syntheses on implementation have primarily focused on factors impacting sustainability, often highlighting the factors required to maintain interventions in the long term rather than those influencing the initial stages of their implementation [31, 35, 37]. However, those are crucial as many interventions are discontinued at these early stages of implementation. Previous reviews suggest that the most important implementation factors related to sustainability can be found at school level and are related to staff and logistical requirements, with strong leadership support and commitment, recognition of potential intervention benefits for both staff and pupils, and flexibility in intervention delivery as key facilitators [31, 35, 37]. Barriers were high staff turnover, competing priorities, and insufficient resources.

The present systematic review builds upon this body of evidence by focusing on barriers and facilitators reported during earlier phases of translational intervention research—specifically during the design and evaluation phases of school-based interventions. Using the Consolidated Framework for Implementation Research (CFIR [33]; see Supplementary Material 2 for an overview of domains), we sought to summarize up-to-date evidence from qualitative research on barriers and facilitators of implementing school-based interventions to prevent mental disorders or promote mental health within secondary schools in high-income countries. Based on these findings, we developed recommendations for the implementation of such interventions and identified evidence gaps to guide future research.

## Methods

This systematic review adheres to standards of the Cochrane Collaboration [38, 39] and is reported in line with the Preferred Reporting Items for Systematic Reviews and Meta-Analyses (PRISMA; [40]; see PRISMA checklist in Supplementary Material 1). Differences between the prospective preregistration on December 15, 2023 (PROSPERO-ID: CRD42023493299; OSF-ID: 10.17605/OSF.IO/7E6PC), and the final review are presented as Supplementary Material 3.

### Search strategy

The search strategy was developed based on related systematic reviews [31, 32, 41, 42] and discussions within the review team, with a relevant number of team members (SKS, KL, MW, DF) being experienced in developing search strategies for systematic reviews. On December 15, 2023, seven electronic databases were searched for eligible primary studies including APA PsycNet (incl. PsycInfo, PsycArticles, PsycExtra), CINAHL, the Cochrane Central Register of Controlled Trials (CENTRAL), Embase, the Education Resources Information Center (ERIC), Scopus, and Web of Science. The search strategy comprised four clusters of search terms related to (a) population and setting (e.g., school* or student*), (b) intervention and program, (c) intervention targets (e.g., mental health, wellbeing, resilience), and (d) factors affecting the implementation (e.g., determinants, barriers). MeSH and Emtree terms were used where applicable. Terms within one cluster were linked using the Boolean operator *OR*, while clusters were combined using the operator *AND* (see Supplementary Material 4 for syntaxes). Moreover, we searched reference lists of eligible primary studies and thematically related systematic reviews [31, 32, 41, 42]. In case we identified potentially eligible randomized-controlled trials (RCTs), we searched for qualitative or mixed methods sister papers reporting on the implementation by means of Google Scholar citation search for a maximal sensitive approach. Furthermore, we also searched for eligible studies citing those included in our review via Google Scholar.

### Selection criteria

Eligible studies were qualitative or mixed methods studies that assessed barriers and/or facilitators of the implementation of interventions aiming at the prevention of mental disorders or the promotion of mental health, wellbeing and resilience in pupils (or students) of regular secondary or high schools in high-income countries according to the classification of the World Bank [43]. Studies were eligible irrespective whether they reported on barriers and/or facilitators in the design or evaluation phase of the intervention. No exclusion criteria were applied in relation to the implementation phase. Preventive interventions referred to structured interventions aiming to reduce the likelihood of future mental disorders and health promotion interventions referring to interventions aiming to promote positive aspects of mental health, wellbeing, and psychological resources [44, 45]. Interventions were eligible irrespective of their format, delivery mode, setting, providers, length, or frequency. Secondary or high schools were those that were intermediate between elementary school and university and usually offer general, technical, vocational and/or pre-college courses [46]. Barriers and facilitators were those factors that were identified as determinants for the implementation of the program, that were either reported to hinder the implementation (i.e., barriers) or promote the implementation (i.e., facilitators). The search was limited to studies published after January 1, 2013, to provide an up-to-date summary of evidence due to rapidly changing educational systems in many high-income countries (e.g., digitalization, changes in learning concepts [47]).

### Study selection

Deduplication was performed in *Zotero* [48]. Title/abstracts and full texts were screened by two reviewers (CS, LR, MC, SoS) independently in *Rayyan* [49]. A pilot screening of 100 records was performed to ensure between-rater agreement indicating sufficient agreement (91%). Following the conventions of Landis and Koch [50], interrater reliability was substantial at title/abstract level (*kappa* = 0.76) and full-text level (*kappa* = 0.62). At both stages of screenings, disagreements were resolved through discussion or by consulting a senior team member (SKS).

## Data extraction

We developed a customized data extraction sheet, which included information on population characteristics (for both study sample and reports on barriers and facilitators), intervention characteristics, study design, and outcomes. The second part of the data extraction sheet was based on the five domains of the CFIR [33]. Moreover, we extracted information on conflicts of interest.

### Quality appraisal

Quality of primary studies was assessed based on the checklist for qualitative studies of the Critical Appraisal Skills Programme (CASP; [51]), which assesses risk of bias from the following domains: (1) validity of study results (i.e., clear research aim, methodological quality, appropriateness of study design, recruitment and data collection, relationship between researchers and participants); (2) study results (i.e., quality of data analyses, clearness of presentation, consideration of ethical issues); (3) helpfulness of the results (i.e., added value). These aspects were amended by a second tool specifically designed for this review project assessing the quality of information on (4) study population; (5) intervention; (6) implementation, and (7) assessment of implementation determinants (i.e., use of a specific framework, clearness of presentation). All assessments were performed independently by two members of the review team (LR, MC, SoS).

### Data synthesis and analysis

Based on the extracted data, we performed a narrative synthesis. First, we summarized study characteristics narratively in tabular form. Subsequently, we performed a synthesis on barriers and facilitators based on the CFIR [33]. Using the CFIR domains, we present a quantitative overview of barriers and facilitators that were reported in primary studies by means of bar charts. If implementation factors were reported as both barriers and facilitators in our evidence base, those were considered for both categories of implementation factors (e.g., counted for the total number of barriers and facilitators). Subsequently, we qualitatively summarized available evidence on barriers and facilitators narratively in tabular form. For this purpose, we present barriers and facilitators identified in primary studies based on the CFIR domains and provide a qualitative summary of the study findings along with information on the number of studies reporting the respective effect.

## Results

### Study selection

**Fig. 1:**
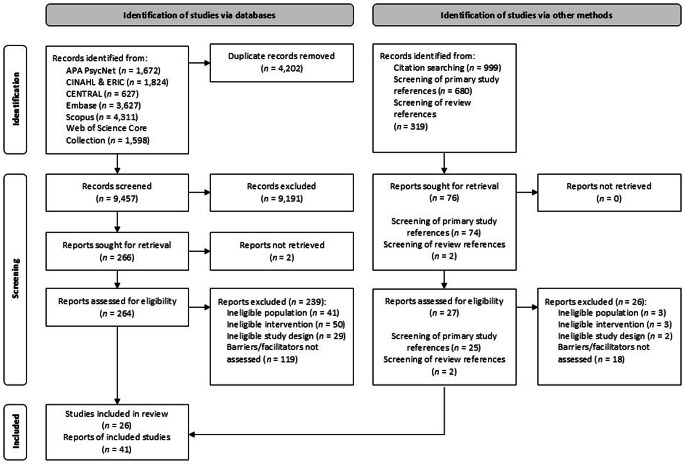
Flow diagram summarizing the study selection process. *Note*. Flow diagram according to the Preferred Reporting Items for Systematic Reviews and Meta-Analyses (PRISMA; [40]). *n* = number of reports/studies

A total of 13,659 eligible records were identified through electronic database searches, with 4,202 duplicates being removed. After deduplication, 9,457 records were screened at title/abstract level, and 266 were assessed at full-text level. Reasons for exclusion at full-text level were missing information on barriers and facilitators of implementation (*n* = 118), ineligible interventions (*n* = 50), study populations (*n* = 41) and study design (*n* = 29). Most studies not reporting on barriers and facilitators of implementation solely reported on implementation outcomes (e.g [52]). Ineligible interventions were most often standard psychotherapy delivered in school settings (e.g [53]). Additionally, 999 records were identified through citation searching, of which one record met the inclusion criteria. Overall, 26 studies met the inclusion criteria (see Fig. 1).

### Characteristics of included studies

Table 1 presents the characteristics of the 26 included studies, with sample sizes ranging from 5 to 8,630 pupils who participated in psychosocial interventions. These studies were published between 2013 and 2023 and were conducted in six Western English-speaking high-income countries including Australia (7 studies; [54–60]), the United States [61–66], the United Kingdom (6 studies each; [67–72]), Canada (4 studies; [73–76]), Ireland (2 studies; [77, 78]), and Scotland (1 study; [79]). Participants were pupils aged between 11 and 19 years, attending grades 4 to 12 in secondary or high schools within different educational systems across countries.

Included studies reported on 24 interventions with heterogenous theoretical backgrounds. Among these, 20 interventions employed a universal approach (i.e., preventative interventions in an unselected pupil population), two used an indicated approach (i.e., targeted a high-risk subgroup of pupils) and four followed a stepped-care model (i.e., offered a preventative intervention as a first step followed by more intensive programs for those who were in need). Group interventions were predominant (19 studies) whereas interventions employed in individual settings were less common (7 studies; [54, 55, 59, 60, 73, 74, 79]). Most interventions were delivered face-to-face (17 studies), while digital-only (6 studies; [54, 55, 59, 60, 74, 78]) and mixed formats (3 studies; [56, 61, 79]) were rarer. A diverse range of professionals facilitated the interventions, including teachers, other school staff, peers, social workers, and clinical psychologists. Although most providers received pre-intervention guidance on the delivery of the intervention, the type and extent of guidance varied considerably.

In terms of study design, 17 studies employed mixed-methods approaches, followed by seven studies employing solely qualitative designs [54, 67, 68, 71–73, 75], one included a cluster randomized controlled trial (cRCT) [60], and one a pre-post design [59]. Both the cRCT and pre-post study reported barriers and facilitators as secondary outcomes providing qualitative data relevant to our review question [59, 60]. Overall, findings on barriers and facilitators were predominantly reported qualitatively in narrative form.

In most studies, diverse stakeholders (e.g., caregivers, school psychologists, school social workers, counsellors) were interviewed to capture implementation factors, with sample sizes ranging from 3 to 296 participants for the assessment of barriers and facilitators. Only in a small number of studies, implementation factors were reported exclusively by specific groups (pupils: 3 studies [57, 59, 60]; teachers: 3 studies [70, 76, 79]; intervention providers: 3 studies [61, 65, 73]).

**Table 1:** Characteristics of included studies

Study ID	Country	*n*_schools_ *n*_pupils_	Intervention population (type; age M ± SD, gender)	Intervention name, background, aim	Setting, Providers	Duration in weeks (sessions), mode	Population reporting on implementation (type; *N*, age M ± SD; gender)	Number of barriers / facilitators reported
Aguilar 2023 [61, 80]	USA	*n*_schools_ = 11 *n*_pupils_ = 6461	pupils (unselected); 14.97 ± 0.92; 51.03% male, 46.21% female, 1.27% trans, 1.50% other	Sources of Strengths, NR, resilience promotion & suicide prevention	Universal group intervention provided by peers and adult advisors with training	1 week (NR), face-to-face and digital	Adult advisors; *n* = 85; NR; NR	*n*_barriers_ = 10 *n*_facilitators_ = 0
McKeague et al. [67, 81]	UK	*n*_schools_ = 10 *n*_pupils_ = 155	pupils (unselected); 17.03 ± 0.77; 18.70% male, 81.30% female	The DISCOVER ‘How to Handle Stress’ workshop, CBT-based, Coping with stress	universal group intervention provided by clinical psychologists with training	2 weeks (2), face-to-face and phone call	participants, nonparticipants & staff; *n* = 34; 21.47(NR); 73.53% female, 26.47% male	*n*_barriers_ = 9 *n*_facilitators_ = 7
Green 2016 [68]	UK	*n*_schools_ = 1 *n*_pupils_ = 5	pupils (selected); NR; 60% male, 40% female	FRIENDS, CBT-based, anxiety prevention & resilience promotion	Indicated group intervention provided by mentors with training	10 weeks (10), face-to-face	Pupils & mentors; *n* = 7; NR; 57.14% female, 42.86% male	*n*_barriers_ = 10 *n*_facilitators_ = 0
Lendrum 2013 [69, 82, 83]	UK	*n*_*schools*_*=* 41 *n*_*pupils*_*=* 8630	pupils (unselected); NR; 48% male, 52% female	Secondary SEAL, based on a model of emotional intelligence, Social & emotional skills promotion	Universal group intervention provided by teachers with training	6 weeks (6), face-to-face	Pupils, diverse school staff, external intervention personnel; NR; NR	*n*_barriers_ = 9 *n*_facilitators_ = 5
Dowling 2020 [77, 84]	Ireland	*n*_*schools*_ *= 32* *n*_*pupils*_ *= 497*	pupils (unselected); *N* = 497; 15.87 (NR); 50.1% males, 49.9% females	MindOut, based on social and emotional learning, wellbeing promotion	Universal group intervention provided by teachers with training	13 weeks (13), face-to-face	Pupils, teachers; *n* = 296; NR; 49.32% female, 50.68% male	*n*_barriers_ = 4 *n*_facilitators_ = 8
de Visser 2020 [70]	UK	*n*_*schools*_*=* 4 *n*_*pupils*_*=* 277	pupils (unselected); NR; 46.57% male, 51.99% female, 1.44% other	‘the sweet-spot’ – intervention, resilience based, drinking prevention	Universal group intervention provided by teachers with training	1 day (1), face-to-face	Teachers; *n* = 4; NR; NR	*n*_barriers_ = 6 *n*_facilitators_ = 0
Wilde 2019 [71]	UK	*n*_*schools*_*=* 7 *n*_*pupils*_*=* NR	pupils (unselected); NR; NR	‘.b’ program, mindfulness & CBT based, mindfulness promotion	Universal group intervention provided by school staff with training	NR (10), face-to-face	School staff; *n* = 78; NR; 46.15% female, 53.85% male	*n*_barriers_ = 11 *n*_facilitators_ = 18
Beames 2023 [54, 85]	Australia	*n*_*schools*_*=* 144 *n*_*pupils*_*=* 6388	pupils (unselected); NR; NR	‘SPARX’ from Future Proofing Program, CBT-based, depression prevention	Universal individual intervention via app	6 (minimum of 4 sessions), digital	School staff; *n* = 23; 40.09 ± 11.82; 73% female, 27% male	*n*_barriers_ = 16 *n*_facilitators_ = 13
O’Dea 2021 [55, 59]	Australia	*n*_*schools*_*=* 4 *n*_*pupils*_*=* 59	pupils (unselected); 14.57 years ± 0.89; 40.68% male, 59.30% female	Smooth Sailing, CBT based, depression and anxiety prevention	Individual stepped-care intervention via web tool and by trained counsellors	6(NR), digital (if required face-to-face)	Counsellors and caregivers; *n* = 10; NR; NR	*n*_barriers_ = 4 *n*_facilitators_ = 5
Dariotis 2023 [62, 86]	USA	*n*_*schools*_*=* 3 *n*_*pupils*_*=* 70	pupils (unselected); 14.70 ± 0.83; 45.70% male, 52.90% female	Mind in Action and Healthy Topics, mindfulness based, NR	Universal group intervention provided by external instructors with training	10(27 to 31), face-to-face	Pupils and observers; *n* = 48; 14.7 ± 0.94 (pupils); 51.1% female, 48.9% male (pupils); 100% male (observers)	*n*_barriers_ = 7 *n*_facilitators_ = 7
Chugani 2022 [63]	USA	*n*_*schools*_*=* 1 *n*_*pupils*_*=* NR	pupils (unselected); NR; NR	DBT STEPS-A, DBT based, mental health symptoms reduction	Universal group intervention provided by health teachers with training	NR (19), face-to-face	school staff; *n* = 23; NR; NR	*n*_barriers_ = 10 *n*_facilitators_ = 0
Ijadi-Maghsoodi 2017 [64]	USA	*n*_*schools*_*=* 2 *n*_*pupils*_*=* 100	pupils (unselected); NR; NR	The Resilience Classroom Curriculum, based on trauma-informed practices, social-emotional learning	Universal group intervention provided by social workers with training	9(9), face-to-face	pupils and social workers; *n* = 29; NR; 57,89% female, 42,11% male (pupils)	*n*_barriers_ = 4 *n*_facilitators_ = 10
Crooks 2022 [73]	Canada	*n*_*schools*_*=* 12 *n*_*pupils*_*=* NR	pupils (unselected); NR; NR	BRISC, transdiagnostic approach for mental health treatment, NR	Universal individual intervention provided by mental health professionals with training	NR(4), face-to-face (digital during COVID-19)	clinicians; *n* = 13; NR; 100% female, 0% male	*n*_barriers_ = 5 *n*_facilitators_ = 15
Hamza 2021 [74, 87]	Canada	*n*_*schools*_*=* 10 *n*_*pupils*_*=* 1884	pupils (unselected); NR; NR	EMPATHY, CBT-based, prevention of substance misuse, depression and anxiety	individual stepped-care intervention provided by resilience coaches with training and digital devices	NR(8 to16); mostly digital but face-to-face contact with resiliency coaches	Resiliency coaches and mental health professionals; *n* = 13; NR; NR	*n*_barriers_ = 23 *n*_facilitators_ = 8
Meixner 2019 [75, 88]	Canada	*n*_*schools*_*=* NR *n*_*pupils*_*=* 41	pupils (selected); 14.87 ± 0.95; NR	IntegraMMA, mindfulness based, self-regulation and academic achievement promotion	Indicated group intervention provided by therapist and teacher with training	18(NR), face-to-face	pupils, therapists, teachers; *n* = 36; 14.87 ± 0.95 (pupils); 20.83% female, 79,17% male (pupils); 70% female, 30% male (teachers)	*n*_barriers_ = 13 *n*_facilitators_ = 8
Bailey 2022 [65]	USA	*n*_*schools*_*=* 10 *n*_*pupils*_*=* 217	pupils (unselected); 15.70 ± NR; 52.10% female	YAM, NR, mental health awareness & resilience promotion	Universal group intervention provided by extension agents with training	3 to 5(5), face-to-face	extension agents (facilitators); *n* = 11; NR; NR	*n*_barriers_ = 7 *n*_facilitators_ = 0
Halliday 2020 [56]	Australia	*n*_*schools*_*=* 1 *n*_*pupils*_*=* 143	pupils (unselected); 14.04 ± 0.28; 59.50% male, 40.50% female	PEPP, based on positive psychology and social-emotional learning, NR	Universal group intervention provided by teachers with training	9(9), face-to-face and digital	teachers, pupils, caregivers, principal; *n* = 152; NR; 62.50% female, 37,50% male (teachers)	*n*_barriers_ = 4 *n*_facilitators_ = 8
Taylor 2014 [72, 89, 90]	UK	*n*_*schools*_*=* 8 *n*_*pupils*_*=* 5030	pupils (unselected); NR; 50.95% male, 49.05% female	Resourceful Adolescent Programme, CBT based, depression prevention	Universal group intervention provided by teachers and external facilitators with training	NR(11), face-to-face	facilitators, teachers and pupils; *n* = 70; NR; 65.71% female, 34,29% male	*n*_barriers_ = 15 *n*_facilitators_ = 4
Goodwin 2023 [78]	Ireland	*n*_*schools*_*=* 10 *n*_*pupils*_*=* 101	pupils (unselected); NR; 12.9% male,80.2% female, 3% other, 4% not specified	Intinn, film based, mental health literacy and resilience promotion	Universal group intervention provided by teachers	1 day(1), digital (but happening in classroom)	Teachers and pupils; *n* = 106; NR; NR	*n*_barriers_ = 7 *n*_facilitators_ = 4
Rickard 2023 [57]	Australia	*n*_*schools*_*=* 2 *n*_*pupils*_*=* 153	pupils (unselected); 15.14 ± NR; 60.13% male, 39.87% female	The Geelong Grammar School positive education program, based on positive psychology, mental health promotion	Universal group intervention provided by teachers with training	NR(NR); face-to-face	pupils; *n* = 33; NR; NR	*n*_barriers_ = 3 *n*_facilitators_ = 1
Exner-Cortens 2020 [76]	Canada	*n*_*schools*_*=* 8 *n*_*pupils*_*=* NR	pupils (unselected); NR; NR	Fourth R, social-emotional learning based, healthy relationships promotion	Universal group intervention provided by teachers with training	NR(NR); face-to-face	teachers; *n* = 90; NR; NR	*n*_barriers_ = 4 *n*_facilitators_ = 0
McAllister 2018 [58, 91]	Australia	*n*_*schools*_*=* 23 *n*_*pupils*_*=* 870	pupils (unselected); 13.00 ± 0.55; 50.34% male, 48.90% female, 0.76% not specified	iCARE-Rural, NR, resilience promotion	Universal group intervention provided by diverse school staff with training	6(6); face-to-face	Facilitators, school staff; *n* = 27; NR; 81.48% female, 18.52% male	*n*_barriers_ = 0 *n*_facilitators_ = 7
O´Dea 2019 [55, 59]	Australia	*n*_*schools*_*=* 4 *n*_*pupils*_*=* 59	pupils (unselected); 14.57 ± 0.89; 40.68% male, 59.30% female	Smooth Sailing, CBT based, depression and anxiety prevention	Individual stepped-care intervention via web tool and by trained counsellors	6(NR), digital (if required face-to-face)	pupils; *n* = 59; 14.57 ± 0.89; 40.68% male, 59.30% female	*n*_barriers_ = 9 *n*_facilitators_ = 0
O´Dea 2021 [60]	Australia	*n*_*schools*_*=* 22 *n*_*pupils*_*=* 1802	pupils (unselected); 14.30 (0.87); 51.60% female	Smooth Sailing, CBT based, depression and anxiety prevention	Individual stepped-care intervention via web tool and by trained counsellors	12(NR), digital (if required face-to-face)	pupils; NR; NR; NR	*n*_barriers_ = 4 *n*_facilitators_ = 0
Lindblom 2018 [66]	USA	*n*_*schools*_*=* 1 *n*_*pupils*_*=* 12	pupils (unselected); 16.00 (NR); 36% male, 64% female	COPE, CBT based, resilience promotion	Universal group intervention provided by counselor with training	7(7); face-to-face	counselor, pupils; *n* = 12; NR; 66.67% female; 33.33% male	*n*_barriers_ = 4 *n*_facilitators_ = 1
Punukollu 2020 [79]	Scotland	*n*_*schools*_*=* 1 *n*_*pupils*_*=* 367	pupils (unselected); NR; NR	SafeSpot, NR, wellbeing and resilience promotion	Universal individual intervention via app supported by teachers and peers	NR(NR); face-to-face and digital	teachers; *n* = 3; NR; NR	*n*_barriers_ = 9 *n*_facilitators_ = 0

Note. NR - not reported

### Quality appraisal

The quality of the included primary studies was evaluated using the CASP checklists for qualitative research (24 studies employing qualitative/mixed methods) and RCTs (1 study), as well as using the assessment tool developed for our review (26 studies). For one study with a pre-post design [59] no suitable tool was available.

**Fig. 2:**
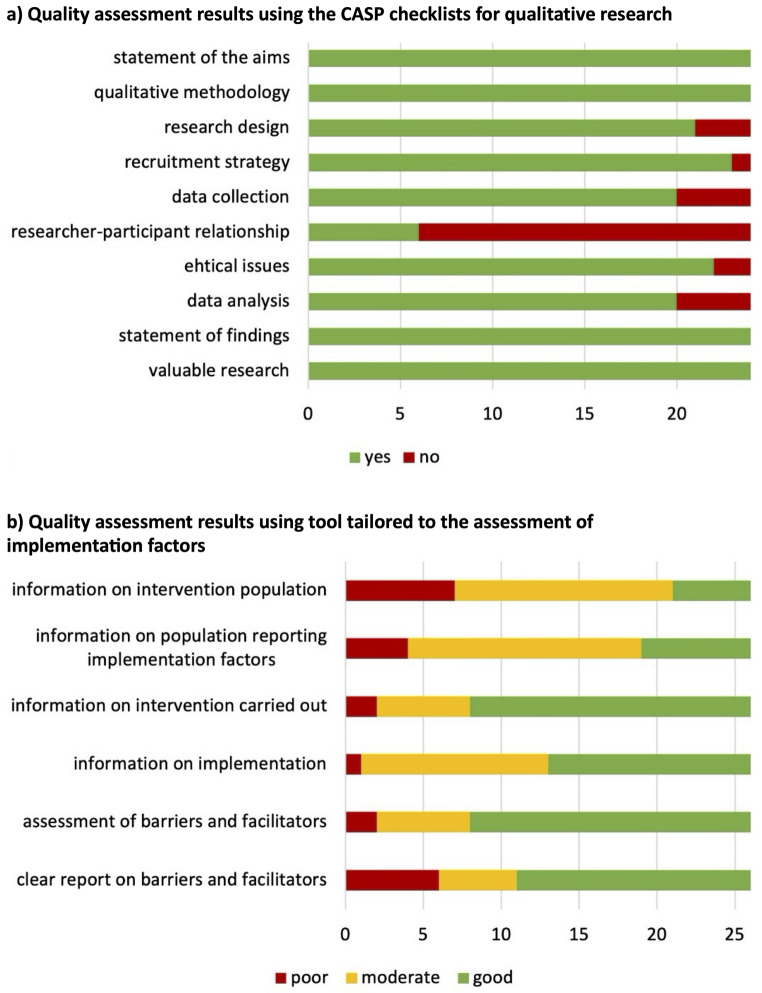
Overview of study quality. *Note*. Quality assessment for **a**) 24 primary studies employing qualitative methods using the CASP checklists for qualitative research and for **b**) 26 primary studies employing the tool developed for our review question

Among the 24 studies assessed using the CASP checklist for qualitative research, all studies clearly defined their research aims, employed appropriate qualitative methodologies, and presented their findings transparently. However, in 18 out of 26 studies the relationship between researchers and participants was insufficiently addressed. While most of the studies employed appropriate recruitment strategies, data collection methods and rigorous data analyses, some displayed minor flaws or did not fully meet the CASP criteria (see Fig. 2). The cRCT [60] met most of the quality criteria of the CASP tool, however, shortcomings were present in the randomization process and blinding of participants.

To assess study quality specifically related to our research question, studies were evaluated using the newly developed tool. More than 50% of the studies provided a comprehensive overview of the implemented interventions. However, many studies lacked detail regarding population characteristics, with only a few studies adequately describing both the pupil population involved in the intervention and those reporting on implementation factors. More than 50% of the studies employed a structured approach or implementation framework to identify implementation factors (see Fig. 2).

### Main findings

**Fig. 3:**
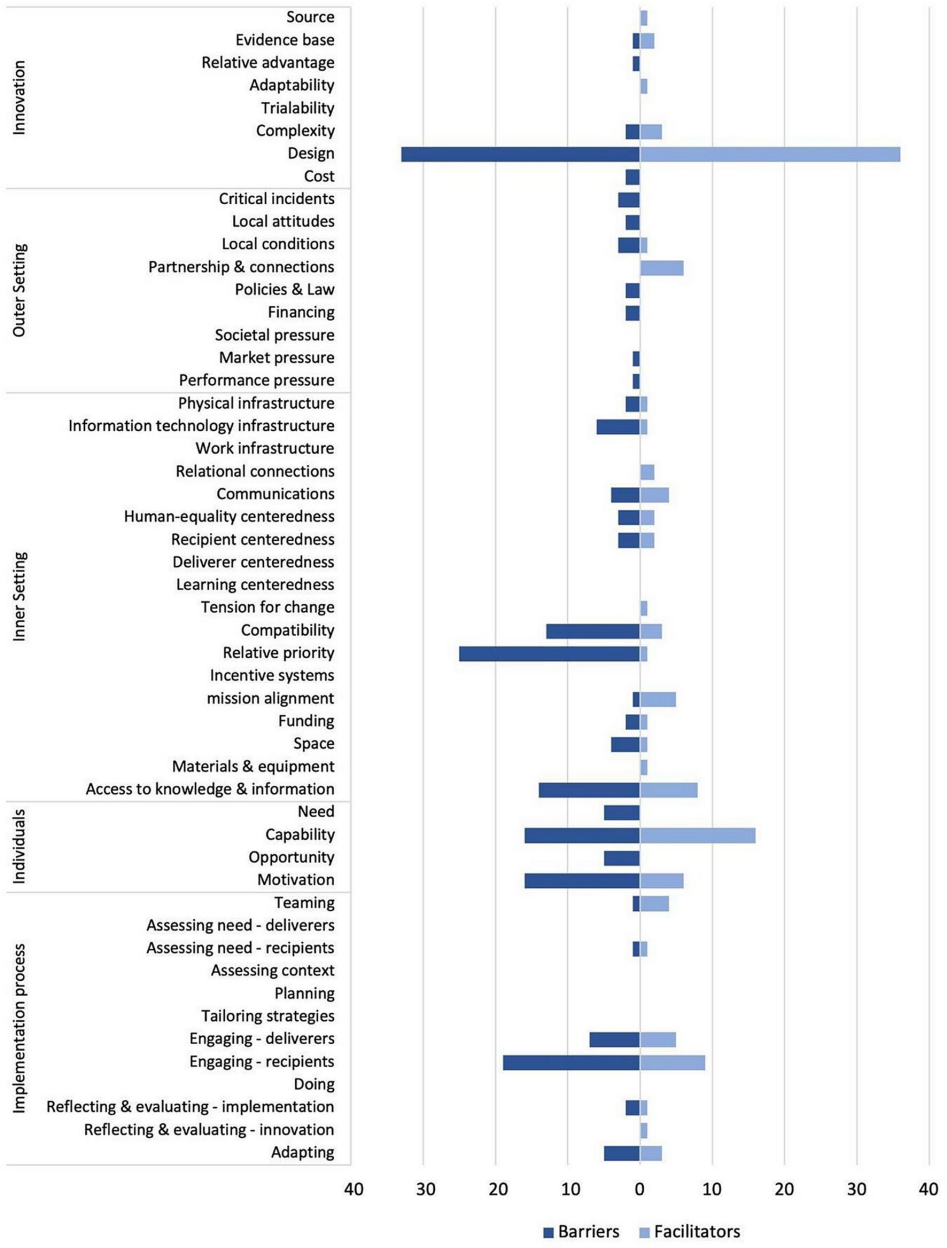
Frequencies of barriers and facilitators reported within Consolidated Framework for Implementation Research domains. *Note*. Please note that the frequencies of barriers and facilitators were calculated based on the total number of implementation determinants identified. In cases where the same determinant was reported as both a barrier and a facilitator in the included studies, it was classified under both categories. Consequently, its frequency was counted twice in the figure

We identified 336 unique implementation factors, of which 207 (61.6%) were classified as barriers and 129 (38.4%) as facilitators of the implementation of school-based preventative mental health interventions. Sixteen studies reported on both barriers and facilitators, while nine studies [59–61, 63, 65, 68, 70, 76, 79] solely focused on barriers, and one study [58] reported only facilitators. The number of distinct implementation factors reported per study ranged from 4 to 31. Figure 3 presents the distribution of barriers and facilitators across the CFIR domains. A tabular presentation of the barriers and facilitators along the CFIR domains can be found in Supplementary Material 6. The most frequently mentioned factors were related to the inner setting (*k* = 110), followed by innovation characteristics (*k* = 82), individuals (*k* = 64), implementation process (*k* = 59), and outer setting (*k* = 21).

Within the ***innovation characteristics domain***, most implementation factors related to the design of mental health interventions. This pattern was observed for both barriers (*k* = 33) and facilitators (*k* = 36). Common design-related barriers included intervention components that were described as too lengthy [62, 66, 70], inappropriate for pupils’ age [69, 74], or outdated [54, 67], leading to participant disengagement. The subgroup of digital interventions was often found to lack engagement and interpersonal relationships, and interventions were perceived as monotonous [56, 59, 74, 79]. In contrast, structured interventions with age-appropriate content relevant to pupils’ daily lives [72, 74, 78], and interactive, pupil-led activities were seen as facilitators [62, 64, 72]. The complexity of the intervention and the time needed for provider training were noted as either barriers or facilitators. Depending on context and providers, the duration of training was considered either too lengthy, and the intervention too complex with an excessive number of components, or, conversely, the flexibility to choose intervention components and the opportunity to acquire additional skills through more time-intensive training were viewed as beneficial [69, 71]. Few studies provided information on factors related to the source of intervention [54], evidence base [54, 71], relative advantage [71], adaptability [54], or costs [71, 72].

Except for five studies [64, 67, 71, 73, 74] reporting on supportive partnerships with external communities and collaborating institutions as facilitators, most ***outer setting*** factors were classified as barriers. Several studies examined face-to-face and digital interventions implemented during the COVID-19 pandemic and indicated that social restrictions, school closures, and changes in program delivery hindered implementation [54, 73, 78]. Additionally, the lack of established mental healthcare infrastructures prevented the transition of services from school-based interventions to standard care in stepped-care programs [74]. Barriers related to policy, legislation, and funding included the discontinuation of funding [74], non-compliance with existing school policies [71], negative attitudes at the local level (e.g., distrust, ambiguous support [56, 74]), and mismatches between community goals and mental health programs [68, 72].

Within the ***inner setting domain***, most implementation factors were reported in the sub-domains of compatibility, relative priority, and access to knowledge and information, again with a focus on barriers. In the relative priority sub-domain, 10 studies reported on conflicts between the intervention and academic obligations—such as negative impacts on exams, grades, and class time [54, 61, 62, 66–68, 70, 74, 75, 78]—making it the most consistently reported barrier. Another common barrier was the lack of time for planning and implementing the intervention in busy school schedules [54, 63, 69, 70, 72, 75]. Only one study mentioned sufficient time and space in school as a facilitator [71]. In the compatibility sub-domain, barriers included rigid curricula [65, 71, 79], poor coordination of school schedules [63, 75, 76], and pressure on teachers to fulfill multiple roles as both intervention providers and educators [65, 71, 79]. By contrast, implementing interventions during school hours [75] and starting programs early in the academic year [77] were identified as facilitators, as they improved compatibility with the school environment. Barriers in the access to knowledge and information sub-domain included limited pre-implementation information [63, 67, 72, 75] and insufficient provider training [56, 63, 66], while clear communication and ongoing training were facilitators [73, 75]. Other facilitators in the inner setting included continuous communication [54, 64], a positive learning climate [64, 75], and a strong commitment to the intervention [67], while common barriers were insufficient space [68], technical issues [54, 55, 59], and poor communication between teachers [54, 63, 69]. None of the studies addressed factors related to work infrastructure, deliverer and learning centeredness, and incentive systems.

In the ***individuals domain***, most factors were reported in the capability and motivation sub-domains, encompassing various stakeholder groups such as pupils, teachers, leaders, and caregivers. Motivation and commitment within these groups could act as either barriers or facilitators [56, 71, 72, 78]. Barriers such as negative group dynamics between pupils, disruptive pupil behavior, and teacher hesitation led to incomplete implementation [54, 62, 74]. In the capability sub-domain, the characteristics and competencies of intervention providers were frequently referred as critical. Providers who were self-efficacious, committed, and attuned to pupils’ needs enhanced implementation and pupil participation [54, 58, 71, 73]. Conversely, when providers were perceived as lacking experience, training, or competence, participation and effective implementation declined [61, 70, 71]. Among pupils, only implementation barriers were noted such as feeling overwhelmed by the intervention [56, 64, 74] or not understanding their roles as peer leaders, if they had to deliver or co-deliver interventions themselves [61, 72]. Other barriers included an unfavorable ratio of pupils to teachers providing the intervention that exceeded their individual capacities, and long waiting times for transferring pupils with mild to moderate symptoms to mainstream health services [74], reflecting a mismatch between needs and available resources [60, 67, 73].

In the ***implementation process domain***, most factors related to recipient and provider engagement, with twice as many barriers than facilitators being reported. Managerial support was both a barrier and facilitator, depending on contextual factors [54, 65, 69, 75]. Successful implementation was aided by staff recruitment, supervisor buy-in, and time allocation for planning [54, 69, 73], while poor management hindered the process [65, 75]. Pupils faced barriers such as limited accessibility, self-referral challenges, and believing the intervention was unnecessary [61, 63, 67, 74]. Additionally, caregiver refusal of consent impeded the implementation, which was related to mental health stigma, lack of communication, and denial of mental health needs [54, 63, 74, 75]. Facilitators included positive perceptions of the intervention, active pupil participation, and external agency involvement [55, 62, 67]. Moreover, teamwork including peer-to-peer learning and input from various stakeholders facilitated the adaptation of the intervention to pupil and provider needs [73]. However, misinformation, conflicting goals, and a lack of cooperation within teams hindered implementation [61].

## Discussion

This review aimed to identify and synthesize multidimensional barriers and facilitators for the implementation of school-based interventions that sought to promote mental health or prevent mental disorders. Based on 26 primary studies, we identified 336 distinctive implementation factors, with barriers (61.6%) outweighing facilitators (34.4%). Using the CFIR [33], we examined barriers and facilitators as individual factors and summarized them within the CFIR domains and sub-domains.

While previous reviews focused on barriers and facilitators to the sustained implementation of mental health interventions in schools in high-income countries [31, 35], and on indicated interventions for adolescents [37], this review advances the existing literature by examining barriers and facilitators also at earlier stages of the translational process and for both school-based mental health interventions targeting prevention and mental health promotion. By encompassing universal, indicated, and stepped-care approaches, this review provides a broader perspective on challenges and opportunities in implementing these interventions. In line with previous reviews [31, 35, 37], our results show that most implementation factors were located within the inner setting. Key barriers in the inner setting included poor compatibility between the intervention and the school environment, insufficient coordination with school schedules, and low priority of mental health interventions due to conflicts with academic commitments. These challenges align with previous research that highlighted logistical issues in schools [37] and competing priorities as major obstacles to implementation [31]. Previous reviews also emphasized that high levels of staff engagement and leadership support are key facilitators [31, 35, 37]. Our findings support this notion by identifying staff and pupil motivation, competence, and experience as key facilitators of the individual domain. Conversely, the lack of these factors and disruptive pupil behavior were significant barriers. The second most frequently identified factor in our review were related to intervention characteristics, particularly intervention design. Structured, age-appropriate, and flexible interventions closely related to pupils’ daily lives were facilitators, while age-inappropriate, lengthy, and outdated components emerged as barriers. Previous research similarly underscored the importance of structured, flexible, and easy-to-implement interventions [31, 37]. While prior reviews focused on factors within the school environment and individual level [31, 35, 37], we additionally examined barriers and facilitators for the implementation process, which had not been studied so far. This gap may partly result from primary studies often describing implementation processes and strategies insufficiently. We found key barriers to include a lack of management support, denial of consent, and limited access to programs, which undermined pupil and staff engagement. Facilitators comprised strong management support, teamwork, stakeholder involvement, staff buy-in, and regular program evaluation and continuous adaptation. As most interventions were still in research phases (i.e., studies on efficacy and effectiveness [92]), implementation factors in the outer setting were mentioned less frequently, both in our review and in previous syntheses [31, 37]. This may reflect that during these phases external factors may be less relevant, as the primary focus is on evaluating the intervention’s efficacy or effectiveness. However, when an intervention proves effective, proper and dissemination studies are conducted, external factors such as funding gaps or unsupportive community polices may become more prominent as barriers to scaling up and sustainable implementation. Consistent with previous research [31, 37], key barriers within this domain included the lack of funding, inadequate infrastructure, and conflicting community priorities. In contrast, supportive partnerships with external organizations and collaborative communities were facilitators. Overall, the barriers and facilitators identified in our review align with previous research [31, 35, 37] and might represent the key barriers and facilitators for school-based mental health interventions.

Our review identified a relative focus on barriers rather than facilitators in primary studies. The predominance of barriers may suggest that researchers are more interested in determinants that hinder successful implementation as they provide potential starting points for implementation strategies that could be used to overcome these barriers. None of the studies reported on implementation strategies to overcome existing barriers and make use of facilitators. Yet, such strategies might rather be described in studies reporting on later phases of the implementation process.

While the CFIR [33] provides a categorization of implementation domains allowing to categorize single and distinct factors—336 factors in case of our review—, their actual impact on implementation is likely to be more complex and interdepended. For instance, one study describes how leadership involvement could have fostered school staff buy-in, with the lack of leadership support acting as a barrier [54]. Simultaneously, they report on capacity issues among providers, which is likely exacerbated by the absence of staff buy-in [54]. Similarly, another study highlights the importance of provider qualities as key facilitators [62]. These attributes led to positive interactions with providers, which, in turn, enhanced pupil engagement, thereby facilitating implementation [62]. Due to this complexity, multi-facetted implementation strategies that combine distinct and single implementation strategies may become prominent avenues to tackle implementation barriers that we identified in this review. However, there were also substantial between-stakeholder differences with respect to some factors. For example, the complexity of an intervention was viewed as a barrier by teachers experiencing increased demands and time constraints, while providers saw it as a facilitator because of the flexibility it offered [69]. These examples illustrate that the relationship between factors is likely multidirectional, with single factors rarely acting independent. Some factors may function as both facilitators and barriers depending on context and evaluator.

Future studies will have to focus on the polyvalence of factors and examine their interplay. Some factors may trigger or inhibit others, suggesting that implementation factors interact rather than accumulate in a linear manner. These complex dynamics were also discussed by previous reviews suggesting that some factors operate across different domains [31] and that implementation is shaped by factors acting at multiple interacting levels [37]. The predominant focus on single factors is common in implementation research but overly simplistic [93], however, to date, comprehensive approaches studying the dynamics and context dependencies of implementation factors such as mediation frameworks [94] or coincident analysis [95] have only been implemented in a small number of primary studies. In our review, none of the primary studies employed such an approach. A larger number of studies using these approaches will allow to derive more mechanistic ideas on implementation and may guide future implementation plans.

### Limitations

For this review, we adhered to the standards for systematic reviews [40, 38] at all stages. Our broad scope allowed to evaluate implementation factors for a large range of school-based preventative mental health interventions. A key strength of this review is the application of the revised CFIR [33], an evidence-based framework for implementation research, to systematically capture and analyze implementation factors.

Several limitations should be considered when interpreting the results. Many of those limitations arise from the included primary studies. First, the evidence base was limited to six Western countries with common language and cultural factors, restricting generalizability of our findings to non-Western high-income countries. Second, in 18 primary studies, the relationship between researcher and participant was not adequately addressed, which could have induced bias, as the researcher’s expectations may shape participants’ responses. Third, reporting standards were relatively low and in many studies barriers and facilitators were reported alongside or conflated with other implementation outcomes such as feasibility and acceptability [59, 65], which may have comprised data quality. This likely results from our review solely including studies that sought to assess implementation determinants during efficacy or effectiveness evaluations. This skew does not reflect a bias in our study selection, but highlights a gap in the literature, as proper implementation evaluations conducted after the establishment of effectiveness are entirely missing and could provide critical insights into barriers and facilitators encountered during the scale-up of interventions. Fourth, the assessment of implementation factors in the primary studies lacked input from relevant stakeholder groups, with external stakeholders and caregivers often been neglected. Also here, later implementation evaluations may provide deeper insights. Moreover, in some studies the populations both receiving the intervention and reporting on implementation factors were described poorly. The exclusion of key stakeholder groups likely results in an unbalanced and underrepresented presentation of perspectives. Fifth, the quality of some studies was compromised by the lack of a structured, evidence-based approaches for assessing implementation factors [59, 60, 63, 65, 66, 68, 76, 79], which might have introduced biases. Sixth, delays between the implementation of the intervention and assessment of implementation factors could have led to recall biases as some interviews were conducted weeks or months after implementation [67, 75, 76].

Other limitations result from our synthesis of evidence. First, our literature search was limited to studies from 2013 to 2023 to reflect recent research on school-based mental health interventions implemented in comparable educational systems [47]. Thereby, we may have missed relevant knowledge from studies published before 2013. Similarly, our focus on high-income countries aimed to reduce between-study differences in educational systems, limiting the applicability of findings to low-resource settings where mental health promotion and prevention are equally important [96]. These decisions were made to limit between-study heterogeneity, yet such differences may still largely impact our results as educational systems are also highly heterogeneous between high-income countries. Some barriers and facilitators might be particularly relevant in specific contexts and might have been overemphasized in our review. Second, we developed a tailored checklist for quality appraisal of primary studies as we found existing checklists [51] to only partly capture relevant aspects of study quality. However, this checklist has not been validated. Third, using the CFIR [33] we employed a structured, evidence-based implementation framework to categorize and analyze implementation factors, yet this approach has its own limitations (see [97] for a critical overview of implementation frameworks and [98, 99] for critical reflections on the CFIR). All steps of data extraction were checked by two review team members, however, in some cases the categorization of implementation factors to CFIR sub-domains could be debated and ratings might have been different for other review teams.

### Implications

Having these limitations in mind, valuable lessons can be drawn from this review for all stages of the translational research process—spanning from science-informed intervention design to dissemination in real-world settings [100].

### Long-term implications on intervention design, evaluation and implementation research

Based on the key factors identified in the **innovation characteristics domain**, future interventions should be developed collaboratively with the target group, providers and relevant stakeholders to ensure acceptability and feasibility of delivery. This aligns with prior literature recommending that intervention curricula should be co-designed with input from target groups [37]. Intervention components should be flexible, allowing pupils to choose from a range of age-appropriate content (e.g., decide between different modules or delivery modes). At the same time, evidence suggests that highly structured and manualized interventions are more likely to be implemented [37], indicating the importance of balancing flexibility with a clear structure for delivery. The development of such interventions might be more expensive at first but is likely to pay off over longer periods.

In terms of **outer and inner settings**, establishing effective communication structures early in the intervention design phase is critical. These structures can include partnerships with external organizations and work towards aligning community goals and infrastructure to support implementation. Establishing supportive measures in the outer setting might be particularly important, as implementation factors in this domain may not only impede but also stop implementation. Previous literature supports this approach, emphasizing that intervention planning and implementation should be carried out by well-structured teams and aligned with relevant policy recommendations to enhance coherence and uptake [37]. To ensure compatibility with the school environment, ongoing communication between leadership, providers, and school staff is essential. As interventions are more difficult to implement if they require a large amount of effort from schools, it may be beneficial to integrate them into the existing school curriculum so that they become a priority alongside other academic obligations. In line with this notion, existing literature emphasizes that shifting priorities in school contexts is a critical challenge for sustainable implementation [31]. Therefore, in the design phase interventions should be developed to fit school timetables. It is also crucial to adapt the intervention to the unique school environments, its culture and the needs of both pupils and teachers.

At the **individual level**, continuous support is needed for intervention providers, which may involve skills training, supervision, and fostering an open culture around mistakes. High-quality training and supervision for intervention providers have been shown to facilitate successful implementation and should therefore be considered a key priority in the implementation of school-based interventions [37]. Pupils should be actively involved in all stages of implementation to motivate their participation. Moreover, pupil participation should be organized in a flexible way, which may also include peer-led activities.

For the **implementation process** ensuring staff buy-in from the early beginning and regularly evaluating and adapting both the intervention and its implementation are key. Involving diverse groups of stakeholders inside and outside the school setting, leveraging their knowledge and experience, likely enriches the implementation process.

Moreover, future studies are needed to gain deeper insights into barriers and facilitators of implementation, especially in the scaling-up phase of school-based mental health interventions when efficacy and effectiveness have already been established. To date, implementation outcomes as well as barriers and facilitators of implementation are only studied in a small number of trials compared to the overall large research effort into the effects of school-based mental health promotion [101–103]. Even in those trials reporting on implementation, the implementation process is often described insufficiently, likely because implementation is not the primary focus. Given the link between implementation and intervention effectiveness [24, 34], and the key role of implementation to establish sustainable interventions [104], it is crucial to transparently report on implementation processes and assess early and late implementation outcomes as part of large-scale effectiveness-implementation hybrid trials in real-world settings [36]. Such trials will benefit from assessing a wide range of potential implementation factors and applying methodological approaches suitable to capture their complex interplay.

### Short-term implications for the implementation of school-based mental health interventions

Beyond informing long-term research agendas, whose results may take years to materialize, the findings of this review can also support more immediate efforts to implement school-based mental health interventions. The large number of implementation factors identified across primary studies (*k* = 336) underscores the substantial complexity faced by schools and intervention providers in real-world settings. Rather than attempting to address all potential barriers and facilitators simultaneously, it is crucial to identify those most relevant to specific school contexts.

To support this, conceptual work conducted within applied settings should aim to further categorize and consolidate the identified factors into higher-order, context-relevant groupings. This practical classification could be developed through structured dialogues with key stakeholders involved in school-based intervention processes.

As highlighted in prior reviews, implementation efforts often face multiple, overlapping barriers [31, 37]. To address these challenges proactively, a previous assessment of anticipated implementation barriers and facilitators may be beneficial. One approach involves using a standardized list (e.g., inspired by Fig. 3) that stakeholders rate based on perceived relevance for the specific context. Implementation strategies can then prioritize the highest-rated factors to tailor support accordingly.

An important unresolved question is whether barriers and facilitators remain consistent across different contexts implementing the same intervention. Deepening our understanding of context specificity will enable the development of more actionable insights from research findings, such as those synthesized in this review. Ultimately, fostering continuous collaboration between researchers and practitioners—and empowering stakeholders to share their implementation experiences beyond the confines of highly structured trials—will be essential for driving meaningful progress [105, 106].

## Conclusion

This review examined the barriers and facilitators to implementing school-based interventions aimed at preventing mental disorders or promoting mental health in secondary schools across Western high-income countries. Findings from 26 primary studies indicate that interventions should be designed to offer flexibility while avoiding excessive complexity for providers. Additionally, effective communication within the school and its broader community context emerged as a critical factor for successful implementation. While adopting these recommendations may initially increase costs related to formative intervention design and ongoing adaptations, such investments are likely to yield long-term benefits. Specifically, they may enhance the sustainability of interventions beyond the lifespan of individual research projects and contribute to the effective promotion of mental health and prevention of mental disorders among pupils in real-world school settings.

## Electronic supplementary material

Below is the link to the electronic supplementary material.

Supplementary Material

## Data Availability

The datasets generated and analyzed in the current review, as well as the review protocol, the screening and extraction guidance and quality assessment tool are available in the OSF repository, https://osf.io/g8n67/.
