# Supplementary material for: Barriers and facilitators for the implementation of preventative mental health interventions among secondary schools in high-income countries: a systematic review
Source: Eur Child Adolesc Psychiatry. 2025 Jun 30;34(12):3713–31. doi: 10.1007/s00787-025-02796-5 (PMC12743069; doi:10.1007/s00787-025-02796-5)
Supplement: Supplementary file 1 — Supplementary Material [file 787_2025_2796_MOESM1_ESM.pdf]

# Supplementary Materials

---

related to **'Barriers and facilitators for the implementation of preventative mental health interventions among secondary schools in high-income countries: A systematic review'**

Sarah K. Schäfer[1,2,*], Sophie Streit[1,*], Christian G. Schäfer[3], Laila B. Roembell[3], Marie Corneli[3], Lea M. Schaubruch[2], Michèle Wessa[2,4,5,6], Klaus Lieb[2,7], Monika Equit[3] & Daniela Fuhr[8,9,10]

1 Clinical Psychology and Psychotherapy for Children and Adolescents, Institue for Psychology, Technische Universität Braunschweig, Braunschweig, Germany

2 Leibniz Institute for Resilience Research, Mainz, Germany

3 Division of Clinical Psychology and Psychotherapy, Department of Psychology, Saarland University, Saarbrücken, Germany

4 DKFZ Hector Cancer Institue at the University Medical Center Mannheim, Mannheim Germany

5 German Cancer Research Center (DKFZ) Heidelberg, Division Cancer Survivorship and Psychological Resilience, Heidelberg, Germany

6 Central Institute of Mental Health, Department of Neuropsychology and Psychological Resilience Research, Mannheim, Germany

7 Department of Psychiatry and Psychotherapy, University Medical Center of Johannes Gutenberg University Mainz, Mainz, Germany

8 Leibniz Institute for Prevention Research and Epidemiology, Bremen, Germany

9 University of Bremen, Health Sciences, Bremen, Germany

10 London School of Hygiene and Tropical Medicine, Department of Health Services Research and Policy, London, United Kingdom

Correspondence related to this Additional File to sarah.schaefer@tu-braunschweig.de.

**Table of Contents**


SM1. PRISMA 2020 checklist. 3

SM2. Domains of the Consolidated Framework for Implementation Research adapted to our research question 7

SM3. Differences between protocol and review 8

SM4. Search strategies per database 12

SM5. Overview of implementation strategies 17

SM6. Summary of barriers and facilitators by CFIR domain 19

References 27

## SM1. PRISMA 2020 checklist.

**Table SM1.** PRISMA 2020 checklist

| Section and Topic | Item # | Checklist item | Location where item is reported |
|---|---|---|---|
| **TITLE** | | | |
| Title | 1 | Identify the report as a systematic review. | Title |
| **ABSTRACT** | | | |
| Abstract | 2 | See the PRISMA 2020 for Abstracts checklist. | Abstract |
| **INTRODUCTION** | | | |
| Rationale | 3 | Describe the rationale for the review in the context of existing knowledge. | Introduction |
| Objectives | 4 | Provide an explicit statement of the objective(s) or question(s) the review addresses. | Introduction |
| **METHODS** | | | |
| Eligibility criteria | 5 | Specify the inclusion and exclusion criteria for the review and how studies were grouped for the syntheses. | Methods, Selection criteria |
| Information sources | 6 | Specify all databases, registers, websites, organisations, reference lists and other sources searched or consulted to identify studies. Specify the date when each source was last searched or consulted. | Methods, Search strategy |
| Search strategy | 7 | Present the full search strategies for all databases, registers and websites, including any filters and limits used. | Methods, Search strategy |
| Selection process | 8 | Specify the methods used to decide whether a study met the inclusion criteria of the review, including how many reviewers screened each record and each report retrieved, whether they worked independently, and if applicable, details of automation tools used in the process. | Methods, Study selection |
| Data collection process | 9 | Specify the methods used to collect data from reports, including how many reviewers collected data from each report, whether they worked independently, any processes for obtaining or confirming data from study investigators, and if applicable, details of automation tools used in the process. | Methods, Data extraction |
| Data items | 10a | List and define all outcomes for which data were sought. Specify whether all results that were compatible with each outcome domain in each study were sought (e.g. for all measures, time points, analyses), and if not, the methods used to decide which results to collect. | Methods, Data extraction |
| | 10b | List and define all other variables for which data were sought (e.g. participant and intervention characteristics, funding sources). Describe any assumptions made about any missing or unclear information. | Methods, Data extraction |
| Study risk of bias assessment | 11 | Specify the methods used to assess risk of bias in the included studies, including details of the tool(s) used, how many reviewers assessed each study and whether they worked independently, and if applicable, details of automation tools used in the process. | Methods, Quality appraisal |
| Effect measures | 12 | Specify for each outcome the effect measure(s) (e.g. risk ratio, mean difference) used in the synthesis or presentation of results. | Not applicable |

| Section and Topic | Item # | Checklist item | Location where item is reported |
|---|---|---|---|
| Synthesis methods | 13a | Describe the processes used to decide which studies were eligible for each synthesis (e.g. tabulating the study intervention characteristics and comparing against the planned groups for each synthesis (item #5)). | Methods, Selection criteria, also see screening guidance via OSF |
| | 13b | Describe any methods required to prepare the data for presentation or synthesis, such as handling of missing summary statistics, or data conversions. | Not applicable |
| | 13c | Describe any methods used to tabulate or visually display results of individual studies and syntheses. | Methods, Data synthesis and analysis |
| | 13d | Describe any methods used to synthesize results and provide a rationale for the choice(s). If meta-analysis was performed, describe the model(s), method(s) to identify the presence and extent of statistical heterogeneity, and software package(s) used. | Methods, Data synthesis and analysis |
| | 13e | Describe any methods used to explore possible causes of heterogeneity among study results (e.g. subgroup analysis, meta-regression). | Not applicable |
| | 13f | Describe any sensitivity analyses conducted to assess robustness of the synthesized results. | Not applicable |
| Reporting bias assessment | 14 | Describe any methods used to assess risk of bias due to missing results in a synthesis (arising from reporting biases). | Not applicable |
| Certainty assessment | 15 | Describe any methods used to assess certainty (or confidence) in the body of evidence for an outcome. | Not applicable |
| **RESULTS** | | | |
| Study selection | 16a | Describe the results of the search and selection process, from the number of records identified in the search to the number of studies included in the review, ideally using a flow diagram. | Results, Study selection,Figure 1 |
| | 16b | Cite studies that might appear to meet the inclusion criteria, but which were excluded, and explain why they were excluded. | Results, Study selection |
| Study characteristics | 17 | Cite each included study and present its characteristics. | Results, Characteristics of included studies, Table 1 |
| Risk of bias in studies | 18 | Present assessments of risk of bias for each included study. | Results, quality appraisal, Figure 2 |

| Section and Topic | Item # | Checklist item | Location where item is reported |
|---|---|---|---|
| Results of individual studies | 19 | For all outcomes, present, for each study: (a) summary statistics for each group (where appropriate) and (b) an effect estimate and its precision (e.g. confidence/credible interval), ideally using structured tables or plots. | Not applicable |
| Results of syntheses | 20a | For each synthesis, briefly summarise the characteristics and risk of bias among contributing studies. | Not applicable |
| | 20b | Present results of all statistical syntheses conducted. If meta-analysis was done, present for each the summary estimate and its precision (e.g. confidence/credible interval) and measures of statistical heterogeneity. If comparing groups, describe the direction of the effect. | Not applicable |
| | 20c | Present results of all investigations of possible causes of heterogeneity among study results. | Not applicable |
| | 20d | Present results of all sensitivity analyses conducted to assess the robustness of the synthesized results. | Not applicable |
| Reporting biases | 21 | Present assessments of risk of bias due to missing results (arising from reporting biases) for each synthesis assessed. | Not applicable |
| Certainty of evidence | 22 | Present assessments of certainty (or confidence) in the body of evidence for each outcome assessed. | Not applicable |
| **DISCUSSION** | | | |
| Discussion | 23a | Provide a general interpretation of the results in the context of other evidence. | Discussion |
| | 23b | Discuss any limitations of the evidence included in the review. | Discussion, Limitations |
| | 23c | Discuss any limitations of the review processes used. | Discussion, Limitations |
| | 23d | Discuss implications of the results for practice, policy, and future research. | Discussion, Implications for the implementation of future interventions |
| **OTHER INFORMATION** | | | |
| Registration and protocol | 24a | Provide registration information for the review, including register name and registration number, or state that the review was not registered. | Methods |
| | 24b | Indicate where the review protocol can be accessed, or state that a protocol was not prepared. | Methods |
| | 24c | Describe and explain any amendments to information provided at registration or in the protocol. | Methods, Supplementary Material 2 |
| Support | 25 | Describe sources of financial or non-financial support for the review, and the role of the funders or sponsors in the review. | Declarations, Funding |
| Competing | 26 | Declare any competing interests of review authors. | Declarations, |

| Section and Topic | Item # | Checklist item | Location where item is reported |
|---|---|---|---|
| interests | | | Competing interests |
| Availability of data, code and other materials | 27 | Report which of the following are publicly available and where they can be found: template data collection forms; data extracted from included studies; data used for all analyses; analytic code; any other materials used in the review. | Declarations, Availability of Data and Materials |

*Note.* From: Page MJ, McKenzie JE, Bossuyt PM, Boutron I, Hoffmann TC, Mulrow CD, et al. The PRISMA 2020 statement: an updated guideline for reporting systematic reviews. BMJ 2021;372:n71. doi: 10.1136/bmj.n71

## SM2. Domains of the Consolidated Framework for Implementation Research adapted to our research question

**Table SM2**. Domains of the Consolidated Framework for Implementation Research (CFIR)

| CFIR domain | Description | Constructs | Applications for the current review |
|---|---|---|---|
| Innovation | "Thing" being implemented (e.g., a new treatment, program or service) | Source, evidence base, relative advantage, adaptability, trialability, complexity, design, costs | Structured psychosocial intervention aiming to prevent mental disorders, promote mental health, wellbeing or resilience |
| Outer setting | Setting in which the inner setting exists, which may have itself multiple levels | Critical incidents, local attitudes, local conditions, partnership & connections, policies & laws, financing, external pressure (i.e., societal pressure, market pressure, performance pressure) | The school district, the educational system as part of the political system |
| Inner setting | Setting in which the innovation is implemented, which may have itself multiple levels | Structural characteristics (i.e., physical, technology and work infrastructure), relational connections, communications, culture (i.e., human equality-centeredness, deliverer-centeredness, learning-centeredness), tension for change, compatibility, relative priority, incentive systems, mission alignment, available resources (i.e., funding, space, materials & equipment), access to knowledge & information | Classes and/or groups within schools |
| Individuals | Roles and characteristics of individuals | High-level leaders, mid-level leaders, opinion leaders, implementation facilitators, implementation leads, implementation team members, other implementation support, innovation recipients, need, capability, opportunity, motivation | Students, caregivers, teachers, other school staff, social workers or psychologists who deliver the intervention incl. their individual values, beliefs, and competences |
| Implementation process | Activities and strategies used to implement the innovation | Teaming, assessing needs (of deliverers or recipients), assessing context, planning, tailoring strategies, engaging (deliverers and recipients), doing, reflecting & evaluating (implementation and innovation), adapting | The processes used for implementing the intervention in the school context including specific implementation strategies (following Powell et al. [1]) |

*Note*. Domains are descripted based on the latest update of the Consolidated Framework for Implementation Research [2]. Adaptations for the purpose of the current review were made by the review team (SoS with supervision of DF and SKS).

## SM3. Differences between protocol and review

**Table SM3.** Differences between protocol and final review (PROSPERO ID: CRD42023493299; OSF ID: 10.17605/OSF.IO/7E6PC)

| | Protocol | Final Review |
|---|---|---|
| Review design | | No changes |
| Review question | | No changes |
| Searches and search strategies | The search strategy for this review will focus on primary studies. Seven electronic databases will be searched from **January 1, 2013, to present**, including APA PsycNet […], CINAHL, Cochrane Central Register of Controlled Trials (CENTRAL), Embase […], ERIC, Scopus, and Web of Science. The strategy will comprise four clusters with search terms related to (a) population and setting (e.g., students, school), (b) interventions and programs, (c) intervention targets (e.g., mental health, wellbeing, resilience), and (d) factors (i.e., determinants, barriers and facilitators) affecting the implementation of those interventions. MeSH terms and Emtree terms will be used where applicable. […] Reference lists of all included studies and thematically related systematic reviews (e.g.,[3–6]) will be checked for eligible primary studies. In case we identify primary outcome papers of randomized controlled trials on eligible psychosocial interventions, we will search for related sister papers, which might report on determinants, facilitators and barriers of intervention implementation by means of Google Scholar citation search. Moreover, we will search Google Scholar for studies citing those studies<br>eligible for our review. | No changes. |
| Types of studies | No changes were made with respect to population(s), intervention(s) and study type(s). | |
| Eligible outcomes | ***Main outcomes:***<br>• Determinants of / factors associated with intervention implementation<br>• Other information on barriers and facilitators of intervention implementation<br>***Additional outcomes:*** | The outcomes were not modified. However, some additional outcomes (e.g. functional status, health service /care use) could not been extracted as the primary studies did not report on these outcomes. Other additional outcomes (i.e., mental health status) were not part of the synthesis. |

| | | |
|---|---|---|
| | • Mental distress (e.g., general mental distress, depressive symptoms, […])<br>• Positive mental health (e.g., wellbeing, life satisfaction, […])<br>• Functional status (e.g., daily functioning, […])<br>• Adverse events<br>• Health service/care use | |
| Data extraction | Two review team members will independently screen titles and abstracts of identified records to assess their eligibility. Irrelevant papers will be excluded immediately. Also at full text level, the eligibility of relevant papers will be checked independently and in duplicate. Any disagreements will be resolved by discussion or by consulting a third reviewer (SKS, KL, DF). We will use *Zotero* to collect and de-duplicate studies. The screening will be performed using *Rayyan* [7]. Inter-rater reliability for both title/abstract and full text screening will be calculated and reported by means of Cohen's kappa. The screening process will be reported in line with the Preferred Reporting Items for Systematic Reviews and Meta-Analyses (PRISMA) flow chart [8].<br>Based on the domains of the **Consolidated Framework for Implementation Research** (CFIR; [2]), we will develop a customized data extraction sheet for the purpose of this review. This has been done in other reviews on facilitators and barriers of intervention implementation [9]. A prefinal version of the data extraction sheet will be uploaded to the Open Science Framework project before the data extraction process will be started. Specifically, the included studies will be coded based on the following aspects:<br>**General information**:<br>• Full citation information<br>• Population and participant characteristics (e.g., sample size, […])<br>• Details on study design<br>• Outcomes, timepoints assessed, and outcome measures | No substantial changes were made. A pilot screening of 100 records was carried out before the initial screening process to ensure substantial agreement between raters.<br><br>The outcomes of each intervention (i.e. the reported quantitative effects of the intervention on mental health, such as means [*M*] and standard deviations [*SD*s], as well as adverse effects) were not extracted as we were not interested in those intervention effects or in the association of implementation outcomes and intervention effects.<br><br>In addition to the implementation factors, the implementation strategies used, and the frequency of their use were coded independently by two raters using the compilation provided by Powell et al. (2015) in the data extraction table. During the review process, we initially planned to report these implementation strategies along with information on their frequency, although this was not specified in our original review protocol. This addition arose because the review forms part of the larger STRESS-Care project, which aims to develop and evaluate a low-intensity stepped-care intervention for German secondary schools. However, we acknowledge that our search strategy was not ideally suited for a synthesis of implementation strategies and that such strategies were not the primary focus of this review. Therefore, we decided not to report on implementation strategies as part of this review. |

| | | |
|---|---|---|
| | • Results (i.e., reported quantitative effects of the intervention on mental health outcomes […])<br>• Adverse outcomes<br>**Information based on the CFIR**:<br>• Intervention: Characteristics of the intervention implemented […]<br>• Outer setting: Economic, political and social context of the organization in which the intervention is implemented<br>• Inner setting: Structural, social and cultural environment of the organization in which the intervention is implemented<br>• Individuals: Characteristics of the individuals involved in the delivery of the intervention […]<br>• Process: Processes used to introduce and maintain an intervention within the organisation […]<br>• Information on the rating of these factors as being either facilitators, irrelevant factors or barriers<br>**Information for quality appraisal**:<br>• Information related to study quality (i.e., statement on study aim, appropriateness of methodology, […])<br>• Potential conflicts of interest (e.g., study authors being involved in the commercial sales of the intervention)<br>Data will be extracted by one reviewer and will be checked by a second reviewer. Any disagreements will be resolved by discussion or by consulting a third reviewer (SKS, KL, DF). The process will be reported in line with the PRISMA standards [8]. | |
| Quality assessment | The study quality will be assessed using the checklist for qualitative studies of the **Critical Appraisal Skills Programme** (CASP; [10]; https://casp-uk.net/checklists/casp-qualitative-studies-checklist-fillable.pdf), assessing risk of bias using the following domains:<br>1. Are the results of the study valid? […]<br>2. What are the results? […]<br>3. Will the results help locally? […]<br>Following the guidelines provided by CASP, we will not calculate an overall quality ratings per study, but report on item-level assessment of study quality based on the checklist. These results will be presented along with the review. Study quality will be rated by two | The quality of the included studies was assessed using the CASP checklists for qualitative research for 24 qualitative or mixed methods studies. In addition, the CASP checklist for RCTs was used to assess the quality of one included cRCT. One study with a pre-post design could not be assessed using the CASP checklists as no suitable tool was available.<br>These quality aspects were amended by items specifically relevant to the review project: quality of information on study population; intervention; implementation, and assessment of implementation determinants (e.g., use of a specific framework, clearness of |

| | | |
|---|---|---|
| | members of the review team independently. Any disagreements will be resolved by discussion or by consulting a third reviewer (SKS, KL, DF). We will not be able the assess the presence of a potential publication bias statistically. However, we will code for each record whether results have been published in a peer-reviewed journal or as part of the 'grey literature' (e.g., dissertations, project reports). Based on this information, we will compare whether results differ substantially by publication type. Such a difference can be interpreted as evidence in favor a publication bias. | presentation). Our quality appraisal tool is available from the related OSF project (https://osf.io/g8n67/).<br>We documented for each record whether the publication was a journal article or a dissertation (part of the grey literature). We found no strong indication for differences between those publication types and performed no further analyses to contrast findings from peer-reviewed journals and the grey literature. |
| Data synthesis | Based on the extracted data, a narrative synthesis of the included studies will be carried out, describing the study population (e.g., descriptive statistics), interventions, measured outcomes and factors examined as potential facilitators or barriers for implementation in text and tabular form.<br>Analyses will follow standards defined by the Cochrane Collaboration for synthesis without meta-analysis (SWiM guidelines; [11]). For single factors, we will follow a vote counting approach and report on all results related to a specific factor (i.e., we will also report on non-significant findings). Results will be<br>presented using the implementation domains suggested by the Consolidated Framework for Implementation Research (CFIR; [2]). | No substantial changes were made. As measured outcomes (i.e., quantitative effects of interventions) were not extracted, they are not reported. In addition to descriptive information on population and intervention characteristics, characteristics of the population reporting on implementation factors (i.e., age, gender, roles) were provided in text form. |

## SM4. Search strategies per database

The search was based on the last 10 years. 01/01/2013 was chosen because some databases always require a search from 01/01 and a search from 12/13, for example, is not possible.

### 1. APA PsycNet (PsycInfo, PsycArticles, PsycExtra)

| # | Query |
|---|---|
| 1 | (student* OR pupil*) in Title, Abstract, Keywords |
| 2 | (secondary OR middle OR high OR comprehensive OR vocational OR technical) AND school*) in Title, Abstract, Keywords |
| 3 | (train* OR program* OR intervention* OR promot* OR prevent* OR service OR communit* OR enhanc* OR increas* OR manag* OR therap* OR treat* OR coach* OR peer*) in Title, Abstract, Keywords |
| 4 | (resilien* OR hardiness OR coping OR "mental health" OR "mental disorder" OR "psychological distress" OR "mental distress" OR "mental burden" OR "psychological burden" OR "mental health literacy") in Title, Abstract, Keywords |
| 5 | (barrier* OR determinant* OR facilitat* OR factor* OR predict* OR enable* OR hinder* OR challenge* ) AND implement*) |
| 6 | (#1 OR #2) AND #3 AND #4 AND #5 from 2013 to 2023 |
| **Search string:** ((title: (barrier*) OR title: (determinant*) OR title: (facilitat*) OR title: (factor*) OR title: (predict*) OR title: (enable*) OR title: (hinder*) OR title: (challenge*)) AND title: (implement*) OR (abstract: (barrier*) OR abstract: (determinant*) OR abstract: (facilitat*) OR abstract: (factor*) OR abstract: (predict*) OR abstract: (enable*) OR abstract: (hinder*) OR abstract: (challenge*)) AND abstract: (implement*) OR (Keywords: (barrier*) OR Keywords: (determinant*) OR Keywords: (facilitat*) OR Keywords: (factor*) OR Keywords: (predict*) OR Keywords: (enable*) OR Keywords: (hinder*) OR Keywords: (challenge*)) AND Keywords: (implement*)) AND ((((((Title:(student*) OR Title:(pupil*) (Title:(secondary) OR Title:(middle) OR Title:(high) OR Title:(comprehensive) OR Title:(vocational) OR Title:(technical)) AND Title:(school*)) OR (Abstract:(secondary) OR Abstract:(middle) OR Abstract:(high) OR Abstract:(comprehensive) OR Abstract:(vocational) OR Abstract:(technical)) AND Abstract:(school*) OR (Subject:(secondary) OR Subject:(middle) OR Subject:(high) OR Subject:(comprehensive) OR Subject:(vocational) OR Subject:(technical)) AND Subject:(school*))) OR (((Title:(student*) OR Title:(pupil*) (Title:(secondary) OR Title:(middle) OR Title:(high) OR Title:(comprehensive) OR Title:(vocational) OR Title:(technical)) AND Title:(school*)) OR (Abstract:(secondary) OR Abstract:(middle) OR Abstract:(high) OR Abstract:(comprehensive) OR Abstract:(vocational) OR Abstract:(technical)) AND Abstract:(school*) OR (Subject:(secondary) OR Subject:(middle) OR Subject:(high) OR Subject:(comprehensive) OR Subject:(vocational) OR Subject:(technical)) AND Subject:(school*))) OR (((title:(student*)) OR (title:(pupil*))) OR ((abstract:(student*)) OR (abstract:(pupil*))) OR ((Subject:(student*)) OR (Subject:(pupil*)))))) AND (((title:(train*)) OR (title:(program*)) OR (title:(intervention*)) OR (title:(promot*)) OR (title:(prevent*)) OR (title:(service)) OR (title:(communit*)) OR (title:(enhanc*)) OR (title:(increas*)) OR (title:(manag*)) OR (title:(therap*)) OR (title:(treat*)) OR (title:(coach*)) OR (title:(peer*))) OR ((abstract:(train*)) OR (abstract:(program*)) OR (abstract:(intervention*)) OR (abstract:(promot*)) OR (abstract:(prevent*)) OR (abstract:(service)) OR (abstract:(communit*)) OR (abstract:(enhanc*)) OR (abstract:(increas*)) OR (abstract:(manag*)) OR (abstract:(therap*)) OR (abstract:(treat*)) OR (abstract:(coach*)) OR (abstract:(peer*))) OR ((Subject:(train*)) OR (Subject:(program*)) OR (Subject:(intervention*)) OR (Subject:(promot*)) OR (Subject:(prevent*)) OR (Subject:(service)) OR (Subject:(communit*)) OR (Subject:(enhanc*)) OR (Subject:(increas*)) OR (Subject:(manag*)) OR (Subject:(therap*)) OR (Subject:(treat*)) OR (Subject:(coach*)) OR | |

| |
|---|
| (Subject:(peer*)))) AND (((((title:("mental disorder")))) OR (((abstract:("mental disorder")))) OR (((Subject:("mental disorder"))))) OR ((((title:("mental health")))) OR (((abstract:("mental health")))) OR (((Subject:("mental health"))))) OR ((((title:(coping imagery)))) OR (((abstract:(coping)))) OR (((Subject:(coping))))) OR ((((title:(hardiness)))) OR (((abstract:(hardiness)))) OR (((Subject:(hardiness))))) OR ((((title:(resilien*)))) OR (((abstract:(resilien*)))) OR (((Subject:(resilien*))))) OR ((((title:("psychological distress")))) OR (((abstract:("psychological distress")))) OR (((Subject:("psychological distress"))))) OR ((((title:("mental distress")))) OR (((abstract:("mental distress")))) OR (((Subject:("mental distress"))))) OR ((((title:("mental burden")))) OR (((abstract:("mental burden")))) OR (((Subject:("mental burden"))))) OR ((((title:("psychological burden")))) OR (((abstract:("psychological burden")))) OR (((Subject:("psychological burden"))))) OR ((((title:("mental health literacy")))) OR (((abstract:("mental health literacy")))) OR (((Subject:("mental health literacy"))))))) *AND* **Year**: 2013 *To* 2023 |

2. **Cumulative Index to Nursing and Allied Health Literature (CINAHL) and Education Resources Information Center (ERIC) via EbscoHost**

| # | **Query** |
|---|---|
| 1 | TI ( student* OR pupil* ) OR AB ( student* OR pupil* ) OR SU ( student* OR pupil* ) |
| 2 | TI ( ( secondary OR middle OR high OR comprehensive OR vocational OR technical ) AND school* ) ) OR AB ( ( secondary OR middle OR high OR comprehensive OR vocational OR technical ) AND school* ) ) OR SU ( ( secondary OR middle OR high OR comprehensive OR vocational OR technical ) AND school* ) ) |
| 3 | TI ( train* OR program* OR intervention* OR promot* OR prevent* OR service OR communit* OR enhanc* OR increas* OR manag* OR therap* OR treat* OR coach* OR peer* ) OR AB ( train* OR program* OR intervention* OR promot* OR prevent* OR service OR communit* OR enhanc* OR increas* OR manag* OR therap* OR treat* OR coach* OR peer* ) OR SU ( train* OR program* OR intervention* OR promot* OR prevent* OR service OR communit* OR enhanc* OR increas* OR manag* OR therap* OR treat* OR coach* OR peer* ) |
| 4 | TI ( resilien* OR hardiness OR coping OR "mental health" OR "mental disorder" OR "psychological distress" OR "mental distress" OR "mental burden" OR "psychological burden" OR "mental health literacy" ) OR AB ( resilien* OR hardiness OR coping OR "mental health" OR "mental disorder" OR "psychological distress" OR "mental distress" OR "mental burden" OR "psychological burden" OR "mental health literacy" ) OR SU ( resilien* OR hardiness OR coping OR "mental health" OR "mental disorder" OR "psychological distress" OR "mental distress" OR "mental burden" OR "psychological burden" OR "mental health literacy" ) |
| 5 | TI ( barrier* OR determinant* OR facilitat* OR factor* OR predict* OR enable* OR hinder* OR challenge* ) AND implement* ) ) OR AB ( barrier* OR determinant* OR facilitat* OR factor* OR predict* OR enable* OR hinder* OR challenge* ) AND implement* ) ) OR SU ( barrier* OR determinant* OR facilitat* OR factor* OR predict* OR enable* OR hinder* OR challenge* ) AND implement* ) ) |
| 6 | (#1 OR #2) AND #3 AND #4 AND #5 from 2013 to 2024 |

### 3. Cochrane Central Register of Controlled Trials (CENTRAL)

| # | Query |
|---|---|
| 1 | student* OR pupil* |
| 2 | (secondary OR middle OR high OR comprehensive OR vocational OR technical) AND school* |
| 3 | MeSH descriptor: [Schools] explode all trees |
| 4 | #1 OR #2 OR #3 |
| 5 | MeSH descriptor: [Psychosocial Intervention] explode all trees |
| 6 | MeSH descriptor: [Health Promotion] explode all trees |
| 7 | MeSH descriptor: [Mental Health Services] explode all trees |
| 8 | MeSH descriptor: [Mentoring] in all MeSH products |
| 9 | train* OR program* OR intervention* OR promot* OR prevent* OR service OR communit* OR enhanc* OR increas* OR manag* OR therap* OR treat* OR coach* OR peer*:ti,ab,kw |
| 10 | #5 OR #6 #7 OR #8 OR #9 |
| 11 | MeSH descriptor: [Emotional Adjustment] explode all trees |
| 12 | MeSH descriptor: [Resilience, Psychological] explode all trees |
| 13 | resilien* OR hardiness OR coping OR "mental health" OR "mental disorder" OR "psychological distress" OR "mental distress" OR "mental burden" OR "psychological burden" OR "mental health literacy":ti,ab,kw |
| 14 | MeSH descriptor: [Stress, Psychological] explode all trees |
| 15 | "mental health literacy":ti,ab,kw |
| 16 | MeSH descriptor: [Health Literacy] explode all trees |
| 17 | #11 OR #12 OR #13 OR #14 OR #15 OR #16 |
| 18 | barrier* OR determinant* OR facilitat* OR factor* OR predict* OR enable* OR hinder* or challenge* |
| 19 | #4 AND #10 AND #17 AND #18 with Publication Year from 2013 to 2023, in Trials |

### 4. Embase via Embase.com including PubMed

| # | Query |
|---|---|
| 1 | student* OR pupil* |
| 2 | (secondary OR middle OR high OR comprehensive OR vocational OR technical) AND school* |
| 3 | 'school'/exp |
| 4 | #1 OR #2 OR #3 |
| 5 | 'psychosocial intervention'/exp |
| 6 | 'health promotion'/exp |
| 7 | 'mental health service'/exp |
| 8 | 'mentoring'/exp |
| 9 | train* OR program* OR intervention* OR promot* OR prevent* OR service OR communit* OR enhanc* OR increas* OR manag* OR therap* OR treat* OR coach* OR peer* |
| 10 | #5 OR #6 OR #7 OR #8 OR #9 |
| 11 | 'psychological adjustment'/exp |
| 12 | 'psychological resilience'/exp |
| 13 | resilien* OR hardiness OR coping OR "mental health" OR "mental disorder" OR "psychological distress" OR "mental distress" OR "mental burden" OR "psychological burden" OR "mental health literacy":ti,ab,kw |
| 14 | 'health literacy'/exp |
| 15 | #11 OR #12 OR #13 OR #14 |

| | |
|---|---|
| 16 | (barrier* OR determinant* OR facilitat* OR factor* OR predict* OR enable* OR hinder* OR challenge*) AND implement*:ti,ab,kw |
| 17 | #4 AND #10 AND #15 AND #16 |
| 18 | #17 AND (2013:py OR 2014:py OR 2015:py OR 2016:py OR 2017:py OR 2018:py OR 2019:py OR 2020:py OR 2021:py OR 2022:py OR 2023:py OR 2024:py) AND ([article]/lim OR [article in press]/lim) AND [english]/lim AND ([embase]/lim OR [medline]/lim OR [embase classic]/lim OR [preprint]/lim OR [pubmed-not-medline]/lim) AND [medline]/lim |

### 5. Scopus.com

| # | **Query** |
|---|---|
| 1 | (student* OR pupil*) in Title, Abstract, Keywords |
| 2 | (secondary OR middle OR high OR comprehensive OR vocational OR technical) AND school*) in Title, Abstract, Keywords |
| 3 | (train* OR program* OR intervention* OR promot* OR prevent* OR service OR communit* OR enhanc* OR increas* OR manag* OR therap* OR treat* OR coach* OR peer*) in Title, Abstract, Keywords |
| 4 | (resilien* OR hardiness OR coping OR "mental health" OR "mental disorder" OR "psychological distress" OR "mental distress" OR "mental burden" OR "psychological burden" OR "mental health literacy") in Title, Abstract, Keywords |
| 5 | (barrier* OR determinant* OR facilitat* OR factor* OR predict* OR enable* OR hinder* OR challenge* ) AND implement*) |
| 6 | (#1 OR #2) AND #3 AND #4 AND #5 from 2013 to 2024 |
| **Search String**: ((TITLE-ABS-KEY ( student* OR pupil* ) OR TITLE-ABS-KEY ( ( secondary OR middle OR high OR comprehensive OR vocational OR technical ) AND school* ) ) ) AND ( TITLE-ABS-KEY ( train* OR program* OR intervention* OR promot* OR prevent* OR service OR communit* OR enhanc* OR increas* OR manag* OR therap* OR treat* OR coach* OR peer* ) ) AND ( TITLE-ABS-KEY ( resilien* OR hardiness OR coping OR "mental health" OR "mental disorder" OR "psychological distress" OR "mental distress" OR "mental burden" OR "psychological burden" OR "mental health literacy" ) ) AND ( TITLE-ABS-KEY ( ( barrier* OR determinant* OR facilitat* OR factor* OR predict* OR enable* OR hinder* OR challenge* ) AND implement* ) ) AND PUBYEAR > 2012 AND PUBYEAR < 2025 AND ( LIMIT-TO ( SUBJAREA , "MEDI" ) OR LIMIT-TO ( SUBJAREA , "SOCI" ) OR LIMIT-TO ( SUBJAREA , "PSYC" ) OR LIMIT-TO ( SUBJAREA , "NURS" ) OR LIMIT-TO ( SUBJAREA , "ENVI" ) OR LIMIT-TO ( SUBJAREA , "HEAL" ) OR LIMIT-TO ( SUBJAREA , "MULT" ) OR LIMIT-TO ( SUBJAREA , "NEUR")) | |

**6. Web of Science**

| # | **Query** |
|---|---|
| 1 | (TS=(student* OR pupil*)) " |
| 2 | (TS=((secondary OR middle OR high OR comprehensive OR vocational OR technical) AND school*)) |
| 3 | #1 OR #2 |
| 4 | (TS=(train* OR program* OR intervention* OR promot* OR prevent* OR service OR communit* OR enhanc* OR increas* OR manag* OR therap* OR treat* OR coach* OR peer*)) |
| 5 | (TS=(resilien* OR hardiness OR coping OR "mental health" OR "mental disorder" OR "psychological distress" OR "mental distress" OR "mental burden" OR "psychological burden" OR "mental health literacy" )) |
| 8 | TS=((barrier* OR determinant* OR facilitat* OR factor* OR predict* OR enable* OR hinder* OR challenge*) AND implement*) |
| 9 | #3 AND #4 AND #5 AND #6 and 2024 or 2023 or 2022 or 2021 or 2020 or 2018 or 2019 or 2017 or 2016 or 2015 or 2014 or 2013 (Publication Years) and Article (Document Types) and Education Educational Research or Psychology or Behavioral Sciences or Health Care Sciences Services or Psychiatry or Public Environmental Occupational Health or Pediatrics or Sociology or Social Sciences Other Topics or Family Studies or Social Issues or Social Work or Neurosciences Neurology or Rehabilitation or Cultural Studies (Research Areas) and English (Languages) |

## SM5. Overview of implementation strategies

**Table SM5.** Implementation strategies that could be reported in primary

| Implementation strategies defined by Powell [12] |
|---|
| Access new funding |
| Alter incentive/allowance structures |
| Alter patient/consumer fees |
| Assess for readiness and identify barriers and facilitators |
| Audit and provide feedback |
| Build a coalition |
| Capture and share local knowledge |
| Centralize technical assistance |
| Change accreditation or membership requirements |
| Change liability laws |
| Change physical structure and equipment |
| Change record systems |
| Change service sites |
| Conduct cyclical small tests of change |
| Conduct educational meetings |
| Conduct educational outreach visits |
| Conduct local consensus discussions |
| Conduct local needs assessment |
| Conduct ongoing training |
| Create a learning collaborative |
| Create new clinical teams |
| Create or change credentialing and/or licensure standards |
| Develop a formal implementation blueprint |
| Develop academic partnerships |
| Develop an implementation glossary |
| Develop and implement tools for quality monitoring |
| Develop and organize quality monitoring systems |
| Develop disincentives |
| Develop educational materials |
| Develop resource sharing agreements |
| Distribute educational materials |
| Facilitate relay of clinical data to providers |
| Facilitation |
| Fund and contract for the clinical innovation |
| Identify and prepare champions |
| Identify early adopters |
| Increase demand |
| Inform local opinion leaders |
| Intervene with patients/consumers to enhance uptake and adherence |
| Involve executive boards |
| Involve patients/consumers and family members |
| Make billing easier |
| Make training dynamic |
| Mandate change |
| Model and simulate change |
| Obtain and use patients/consumers and family feedback |

| Implementation strategies defined by Powell [12] |
| --- |
| Obtain formal commitments |
| Organize clinician implementation team meetings |
| Place innovation on fee for service lists/formularies |
| Prepare patients/consumers to be active participants |
| Promote adaptability |
| Promote network weaving |
| Provide local technical assistance |
| Provide ongoing consultation |
| Provide clinical supervision |
| Purposely reexamine the implementation |
| Recruit, designate, and train for leadership |
| Remind clinicians |
| Revise professional roles |
| Shadow other experts |
| Stage implementation scale up |
| Start a dissemination organization |
| Tailor strategies |
| Use advisory boards and workgroups |
| Use an implementation advisor |
| Use capitated payments |
| Use data experts |
| Use data warehousing techniques |
| Use mass media |
| Use other payment schemes |
| Use train-the-trainer strategies |
| Visit other sites |
| Work with educational institutions |

***Note***. Definitions of the respective implementation strategies can be found in Powell [12]. We found no evidence in the primary studies, that implementation strategies were used to exploit facilitators or to overcome barriers.

## SM6. Summary of barriers and facilitators by CFIR domain

**Table SM6.** Barriers and Facilitators for the implementation of school-based interventions to prevent mental disorders or promote mental health in secondary schools categorized based on the Consolidated Framework for Implementation Research

| CFIR domains | Subdomain(s) | Barriers | Facilitators |
|---|---|---|---|
| Innovation | Source | NR | Trust and reputation of the provider [13] |
| Innovation | Evidence base | Worries about insufficient evidence [14] | Provision of evidence [13,14] |
| Innovation | Relative advantage | Worries about adverse effects [14] | NR |
| Innovation | Adaptability | NR | Flexibility of intervention delivery [13] |
| Innovation | Trialability | NR | NR |
| Innovation | Complexity | High complexity of the intervention (e.g. encompassing too many components), multiple models of implementation [15]; time-consuming training for intervention providers [14] | High complexity of the intervention, multiple models of implementation (e.g. providers freedom to decide which components to implement) [15]; time-consuming training of additional teacher skills [14]; clear instruction on service use provided by research team [16] |
| Innovation | Design | Session duration was either too short to cover all materials [17–19] or too long, making it difficult for students to stay focused [20–23]; age-inappropriate materials [15,20,22,24,25]; digital components consume too much data volume, were perceived as boring, not sufficiently engaging, could not compensate interpersonal relationships [21,24–26]; outdated technique, content, gamification and graphics, problems with usability, lack of personalization, repetitive and theoretical [13,20,23,27]; small group sizes making activities challenging, discomfort with physical activities or role plays within the group [28–30]; overemphasis on text-based and sit-down activities | Structured, client-driven, manualized programs easy to deliver [18,32]; adequate intervention frequency (e.g. one session per week), caseload and workload [16,26,27]; rich intervention material (e.g. booklets, posters, presentations) and techniques [26,27,33]; content relevant to daily life, appropriate communication on mental health, provision of useful strategies (e.g. problem solving), insights into mental health topics [22–24]; clear intervention content, purpose of activities helped to remember the session [14,17]; interactive, student-led activities and games promoted student engagement [17,22,31]; group environment counteracts stereotypes, increases comfort and offers the opportunity to meet new people [29]; personalization |

| | | | |
|---|---|---|---|
| | | over interactive components [25,27,31]; screening questions were perceived as confusing and not engaging [16,23]; content not culturally sensitive [24]; distraction through food offers [17] | and age-appropriateness [13,27,33]; user-friendly gamified app on students' devices, web-based psychoeducation [13,16,24]; data-driven assessment tools improved implementation and enhanced staff capacity to meet students' needs [13,32] |
| **Outer setting** | Cost | High costs of training for intervention deliverers [14]; intervention not sustainable due to high costs [22] | NR |
| | Critical incidents | COVID-19-pandemic: social distancing and school closures required program adaptions (virtual/remote) [13,23,32]; too much screen-time due to online learning [23] | NR |
| | Local attitudes | Transgenerational distrust in the government hindered participation in online intervention [24]; ambiguous support of schools by the ministry [26] | NR |
| | Local conditions | Gap in services for youth under 18 years with mild to moderate symptoms [24]; linkage services (e.g. transportation) as barrier to accessing treatment [24]; intervention not assigned the same importance as school subjects by government [22] | Continued support of students after implementation outside of the program [24] |
| | Partnerships & connections | NR | External agency as providers for mental health support [27]; seamless referral to community partners [32]; cooperation with other schools [14]; academic-community partnership within the school district as a foundation for tailoring and delivering the curriculum [31,32] |
| | Policies & law | Conflict with existing "no-phone" school policies; high demands on schools to adapt standards in key curriculum areas and respond rapidly to policy changes [14] | NR |
| | Financing | Only temporary financial support, end of funding with termination of research program [24]; unexpected expenses for intervention materials (e.g. snacks, posters) and travel costs [34] | NR |

| | | | | |
|---|---|---|---|---|
| Inner setting | External pressure | Societal pressure | NR | NR |
| | | Market pressure | Conflict between program objectives and an ongoing construction project, which negatively affected room availability [28] | NR |
| | | Performance pressure | Governmental pressure on schools to deliver mental health support [25] | NR |
| | Structural characteristics | Physical infrastructure | Poor room availability due to construction works [28]; location too small for group size [18] | School setting as familiar, comfortable and safe surrounding [27] |
| | | Information technology infrastructure | Poor implementation of the room booking system resulting in last minute room changes [28]; low accessibility of technology infrastructure in rural areas and communities with low socioeconomic status [13]; technical barriers with program accessibility, email system and internet connectivity [16,21,35]; problems with survey delivery [34] | Email system used to notify counsellors was valuable [16] |
| | | Work infrastructure | NR | NR |
| | Relational connections | | NR | Staff involvement from the early start of the implementation [15]; already available support structures [14] |
| | Communications | | Lack of communication between providers and/or staff members involved in planning and delivering the intervention [13,15,36]; intervention is presented in a way staff it feels like an "add-on" task [15] | Program presented in a way that the staff feels it can be integrated into the regular curriculum [15]; on-going communication between teachers, administrators and providers with clear allocations of responsibilities and task-sharing [13,31]; sharing intervention experiences with colleagues [14] |
| | Culture | Human-equality centeredness | Issues with representation, language, participation of minority groups and cultural diversity [13,37]; values of teachers, school administrators and caregivers not in line with the intervention content [19] | Establishing structures and rules to keep a positive, non-judgmental climate [29,31] |

| | | | |
|---|---|---|---|
| | Recipient-centeredness | Pre-existing class dynamics (e.g. lack of classroom management, poor school attendance, disruptive behavior) [31]; presence of mental health stigma, low mental health literacy, visibly recruiting students from class to attend the intervention [13,29] | Intervention in line with school values in terms of student welfare and care [27]; viewing all young people as deserving support regardless of context and actions [38] |
| | Deliverer-centeredness | NR | NR |
| | Learning-centeredness | NR | NR |
| Tension for change | | NR | Intervention addressing unmet needs, appropriateness for students' needs [13] |
| Compatibility | | Busy school environment in terms of time frames for delivery and communication between teachers and providers, difficulties in coordinating multiple schedules, infrequent intervention scheduling [22,29,30,33,36]; pressure on teachers to fulfil multiple roles in promoting students' academic success and mental health, lack of time for the staff [14,25,34]; inflexible school curricula [14]; problems with scheduling due to travel [34]; students did not completed homework in health subjects [36]; other external projects and groups were scheduled during intervention time [30] | Compatibility with the school context [13]; program delivery within school hours to enhance access for students [29]; starting the program early in the academic year as the academic year gets busier throughout the year [33] |
| Relative priority | | Conflict between intervention and academic obligations, including negative impact on grades, impeding exam season, disruptions to normal lessons, conflicts with extracurricular activities [13,18,19,23,24,27–29,37,39]; teachers struggling with time constraints, prioritizing the intervention, protecting time for planning and ongoing communication in school day [13,15,19,22,29,36]; | Sufficient staff time, reserved space in the curriculum, time to embed intervention within the school [14] |

| | | |
|---|---|---|
| | teachers discouraging students from participating in the program due to academic obligations [17,37]; holiday breaks [28]; students with academic difficulties lose learning time due to their participation [24]; difficulties with peer leaders during exam season due to academic obligations [37] | |
| Incentive systems | NR | NR |
| Mission alignment | Mental health not being a priority in school [13] | Commitment to the intervention within school [14]; curriculum responsive school needs [31]; establishing the intervention as part of the school culture, preparing the community for change [14,26]; understanding, that commitment is required [14] |
| Available resources: Funding | Little or no financial resources for implementation [13,15] | Financial resources for staff training and ongoing support [14] |
| Available resources: Space | Shortage of available classrooms [27], sharing facilities with other scheduled groups [29], disruptions, room changes due to other school events [17], surrounding noise [29] | Appropriate physical space that is private, comfortable in terms of temperature, provides equipment that facilitates learning [29] |
| Available resources: Materials & equipment | NR | Provision of high-quality resources (WLAN, devices) by the research team [13] |
| Access to knowledge & information | Lack of information about the intervention before and during implementation [22,27,29,36]; inadequate or minimal training provided for providers [18,26,36]; difficulties with timely training for team members, inability to train all members at once, supervisors were trained last [32]; lack of ongoing guidance, support or supervision [25,36,37] | Clear communication about who the program was for, ensuring providers and staff have a solid understanding of the intervention [29,32]; importance of training to be comfortable with intervention contents [32,33]; intermittent, ongoing training opportunities, communication with educators, communication about student progress [29,32]; Developing a shared language about the intervention within the school [14] |

| | | | |
|---|---|---|---|
| **individuals** characteristics | Need | Mismatch with recipients' needs (e.g., intervention needs, needs with respect to complexity [27,32,35]); students feeling to down to use the service [21]; waiting time for additional treatment for students with mild to moderate symptoms [24] | NR |
| | Capability | Lack of confidence, skills, expertise, qualification or capacity to implement intervention as school staff were trained poorly [14,19,33,37]; varying quality of delivery [22,28]; students faced challenges engaging with program activities, difficulties using technology, and completing tasks like gratitude letters [24,26,31]; peer leaders misunderstood the intervention goal, teachers experiences role conflicts [19,22,37]; staff turnover, changes in leadership resulting in rapid loss of expertise and capacity [14]; relationship building between external providers and students was difficult over a short time period [22] | Supportive leadership, teams, involving individuals with capacity and expertise [13,14,32,38]; provider qualities: building relationships with students, self-efficacy, engaging personality, being aware of students' needs, ability to reflect on practice, self-awareness and self-disclosure, flexibility [14,17,22,32,38]; teachers being engaging, trusted by students, and experienced in classroom management and education [20] |
| | Opportunity | High caseloads and limited capacity of providers and teachers led to delays in care [13,21,24,25,35] | NR |
| | Motivation | Teachers' and students' enthusiasm, interest, and commitment diminished over time [14,22,23]; lack of motivation, openness, willingness to change [15,21,25,35]; disruptive student behavior, negative group dynamics including interrupting and disrespecting the provider, off-task behaviors, refusing task completion, teachers hesitation and opposition to intervention process, caregivers having other priorities [13,17,24,33]; not valuing the program or taking it seriously [33,37]; staff worries that approach is insufficient for change [13] | Enthusiasm and commitment at leadership level [14,15,26]; motivation for staff-buy-in [13]; perception that the intervention was viewed as an effective practice and was experienced as a needed improvement [26,32]; support for the intervention by caregivers [26] |

| | | | |
|---|---|---|---|
| Implementation process | Teaming | Issues with communication among providers, having conflicting ideas within the team about which activities to implement [37] | Benefits of offering individual and group supervision facilitating peer-to-peer learning and focusing on students' needs [32]; combination of formal and informal structure that supported the implementation [32]; valuable input from multiple stakeholders, students involved in decision making [26] |
| | Assessing need - deliverers | NR | NR |
| | Assessing need – recipients | Selection of participants by teachers based on observed behavior [28] | Mental health counsellors assessing youth based on individual needs [24] |
| | Assessing context | NR | NR |
| | Planning | NR | NR |
| | Tailoring strategies | NR | NR |
| | Engaging: Innovation deliverers | Lack of leadership and administrative support [15,29,34]; caregiver consent affected by the level of interest from school administrators and teachers as well as and seasonal factors [34]; teachers' mixed attitudes towards the intervention and relationship with providers [22] | Support and involvement from leadership with staff recruitment and allocation of time for planning and preparation [13,15]; teachers' enthusiasm, willingness and involvement in the implementation process [14,26]; supervisor buy-in [32] |
| | Engaging: Innovation recipients | Difficulties with student and teacher buy-in due to limited accessibility, believing the intervention is unnecessary, self-referral to intervention, unawareness of obligatory caregiver involvement and discomfort with sharing feelings in the classroom [16,24,27,36,37]; lack of caregiver consent due to poor marketing and communication about intervention, family dynamics, fear of stigma, obligatory caregiver involvement [13,24,29,36]; worries about privacy and confidentiality [21,24]; contacting caregivers hindered student progress [24] | Intervention actively sought student participation through addressing students' interests or sending text messages [17,27]; students were more interested when the intervention was presented by external institutions [16]; positive perception of the intervention as beneficial for both staff and student well-being, improving mental health, academic attainment, promoting development [14]; supportive environment and relationships because of teacher engagement [24]; caregivers attended appointments to ensure their adolescents had access to mental health care [24] |
| | Doing | NR | Measurement-based care allowed supervisors to monitor implementation fidelity and quality [32] |

| | | | |
|---|---|---|---|
| | Reflecting & evaluating – implementation | Teachers' implementation performance was not evaluated [36]; difficulties with standardizing processes into an implementation protocol due to heterogeneous approaches, techniques and preferences of providers [24] | NR |
| | Reflecting & evaluating – innovation | NR | Getting feedback from teachers about issues with implementing group content in classroom (e.g. disruptive student behavior) [31] |
| | Adapting | Lack of caregiver involvement through intervention process as it depended on capacity and student contribution [28]; content adaptations leading to additional time pressure and workload [25]; poor implementation adaptation based on students' and settings' needs [36]; some provider were misinformed, had differing version of the intervention or conflicting goals [14,28] | Teachers integrating content of intervention into the regular curriculum; continuously improving and adapting the intervention to fit the school community; employing innovative modifications for intervention components that student found difficult [31] |

*Note.* The Roles section of the CFIR outlines applicable roles within the inner and/or outer Setting [2]. In our review, this section was solely used for this purpose, so no barriers or facilitators were coded here in this table. The term "provider" refers to anyone leading or delivering an intervention. NR - not reported.

## References


1. Powell BJ, Waltz TJ, Chinman MJ, Damschroder LJ, Smith JL, Matthieu MM, et al. A refined compilation of implementation strategies: results from the Expert Recommendations for Implementing Change (ERIC) project. Implementation Science. 2015;10:21. doi: 10.1186/s13012-015-0209-1.

2. Damschroder LJ, Reardon CM, Widerquist MAO, Lowery J. The updated Consolidated Framework for Implementation Research based on user feedback. Implementation Science. 2022;17:75. doi: 10.1186/s13012-022-01245-0.

3. Mackenzie K, Williams C. Universal, school-based interventions to promote mental and emotional well-being: what is being done in the UK and does it work? A systematic review. BMJ Open. 2018;8:e022560. doi: 10.1136/bmjopen-2018-022560.

4. March A, Stapley E, Hayes D, Town R, Deighton J. Barriers and Facilitators to Sustaining School-Based Mental Health and Wellbeing Interventions: A Systematic Review. International Journal of Environment Research and Public Health. 2022;19:3587. doi: 10.3390/ijerph19063587.

5. O'Connor CA, Dyson J, Cowdell F, Watson R. Do universal school-based mental health promotion programmes improve the mental health and emotional wellbeing of young people? A literature review. Journal of Clinical Nursing. 2018;27:e412-26. doi: 10.111/jocn.14078.

6. O'Reilly M, Svirydzenka N, Adams S, Dogra N. Review of mental health promotion interventions in schools. Social Psychiatry Epidemiology. 2018;53:647–62. doi:10.1007/s00127-018-1530-1.

7. Ouzzani M, Hammady H, Fedorowicz Z, Elmagarmid A. Rayyan—a web and mobile app for systematic reviews. Systematic Reviews. 2016;5:210. doi:10.1186/s13643-016-0384-4

8. Page MJ, McKenzie JE, Bossuyt PM, Boutron I, Hoffmann TC, Mulrow CD, et al. The PRISMA 2020 statement: an updated guideline for reporting systematic reviews. BMJ. 2021;n71. doi: 10.1136/bmj.n71.

9. Esponda GM, Hartman S, Qureshi O, Sadler E, Cohen A, Kakuma R. Barriers and facilitators of mental health programmes in primary care in low-income and middle-income countries. The Lancet Psychiatry. 2020;7:78–92. doi: 10.1016/S2215-0366(19)30125-7.

10. Singh J. Critical appraisal skills programme. Journal of Pharmacology and Pharmacotherapeutics. 2013;4:76–7. doi: 10.4103/0976-500X.107697.

11. Campbell M, McKenzie JE, Sowden A, Katikireddi SV, Brennan SE, Ellis S, et al. Synthesis without meta-analysis (SWiM) in systematic reviews: reporting guideline. BMJ. 2020;l6890. doi: 10.1136/bmj.l6890.

12. Powell BJ, Waltz TJ, Chinman MJ, Damschroder LJ, Smith JL, Matthieu MM, et al. A refined compilation of implementation strategies: results from the Expert Recommendations for Implementing Change (ERIC) project. Implementation Science. 2015;10:21. doi: 10.1186/s13012-015-0209-1.

13. Beames JR, Werner-Seidler A, Hodgins M, Brown L, Fujimoto H, Bartholomew A, et al. Implementing a Digital Depression Prevention Program in Australian Secondary Schools: Cross-Sectional Qualitative Study. JMIR Pediatrics and Parenting. 2023;6. doi:10.2196/42349.

14. Wilde S, Sonley A, Crane C, Ford T, Raja A, Robson J, et al. Mindfulness training in UK secondary schools: A multiple case study approach to identification of cornerstones of implementation. Mindfulness. 2019;10:376–89. doi: 10.1007/s12671-018-0982-4.

15. Lendrum A, Humphrey N, Wigelsworth M. Social and emotional aspects of learning (SEAL) for secondary schools: Implementation difficulties and their implications for school-based mental health promotion. Child and Adolescent Mental Health. 2013;18:158–64. doi: 10.1111/camh.12006.

16. O'Dea B, King C, Achilles MR, Calear AL, Subotic-Kerry M. Delivering A Digital Mental Health Service in Australian Secondary Schools: Understanding School Counsellors' and Parents' Experiences. Health Services Insights. 2021;14. doi:10.1177/11786329211017689.

17. Dariotis JK, Mabisi K, Jackson-Gordon R, Yang N, Rose EJ, Mendelson T, et al. Implementing Adolescent Wellbeing and Health Programs in Schools: Insights from a Mixed Methods and Multiple Informant Study. Prev Science. 2023;24:663–75. doi: 10.1007/s11121-022-01481-2.

18. Lindblom JR. Cope: Evaluation of a school-based intervention to improve the overall mental health, resiliency, and social-emotional development of rural North Dakota adolescent youth. 2018;78.

19. de Visser RO, Graber R, Abraham C, Hart A, Memon A. Resilience-based alcohol education: Developing an intervention, evaluating feasibility and barriers to implementation using mixed-methods. Health Education Research. 2020;35:123–33. doi:10.1093/her/cyaa006.

20. Rickard NS, Chin TC, Cross D, Hattie J, Vella-Brodrick DA. Effects of a positive education programme on secondary school students' mental health and wellbeing; challenges of the school context. Oxford Review of Education. 2023; 309-331. doi: 10.1080/03054985.2023.2211254.

21. O'Dea B, King C, Subotic-Kerry M, Achilles MR, Cockayne N, Christensen H. Smooth sailing: A pilot study of an online, school-based, mental health service for depression and anxiety. Frontiers in Psychiatry. 2019;10:547. doi: 10.3389/fpsyt.2019.00574.

22. Taylor JA, Phillips R, Cook E, Georgiou L, Stallard P, Sayal K. A qualitative process evaluation of classroom-based cognitive behaviour therapy to reduce adolescent depression. Int. J. Environ. Res. Public Health. 2014;11:5951–69. doi:10.3390/ijerph110605951.

23. Goodwin J, Behan L, Saab MM, O'Brien N, O'Donovan A, Hawkins A, et al. A film-based intervention (Intinn) to enhance adolescent mental health literacy and well-being: multi-methods evaluation study. Mental Health Review Journal. 2023. doi:10.1108/MHRJ-05-2023-0027.

24. Hamza DM, Greenshaw AJ, Hamza SM, Silverstone PH. Qualitative findings from administrators of the EMPATHY (Empowering a multimodal pathway toward healthy youth) programme using the SBIRT framework. British Journal of Guidance & Counselling. 2021;49:533–52. doi:10.1080/03069885.2019.1686121.

25. Punukollu M, Burns C, Marques M. Effectiveness of a pilot school-based intervention on improving scottish students' mental health: a mixed methods evaluation. International Journal of Adolescence and Youth. 2020;25:505–18. doi: 10.1080/02673843.2019.1674167.

26. Halliday AJ, Kern ML, Garrett DK, Turnbull DA. Understanding factors affecting positive education in practice: An Australian case study. Contemporary School Psychology 2020;24:128–45. doi: 10.1007/s40688-019-00229-0.

27. McKeague L, Morant N, Blackshaw E, Brown JSL. Exploring the feasibility and acceptability of a school-based self-referral intervention for emotional difficulties in older adolescents: Qualitative perspectives from students and school staff. Child and Adolescent Mental Health. 2018;23:198–205. doi: 10.1111/camh.12234.

28. Green SL, Atkinson S. Implementation Issues: A 'FRIENDS for life' course in a mainstream secondary school. Educational Psychology in Practice. 2016;32:217–30. doi: 10.1080/02667363.2023.2274028.

29. Meixner T, Irwin A, Wolfe Miscio M, Cox M, Woon S, McKeough T, et al. Delivery of Integra Mindfulness Martial Arts in the Secondary School Setting: Factors that Support Successful Implementation and Strategies for Navigating Implementation Challenges. School Mental Health. 2019;11:549–61. doi:10.1007/s12310-018-9301-4.

30. Exner-Cortens D, Spiric V, Crooks C, Syeda M, Wells L. Predictors of healthy youth relationships program implementation in a sample of Canadian middle school teachers. Canadian Journal of School Psychology. 2020;35:100–22. doi: 10.1177/0829573519857422.

31. Ijadi-Maghsoodi R, Marlotte L, Garcia E, Aralis H, Lester P, Escudero P, et al. Adapting and implementing a school-based resilience-building curriculum among low-income racial and ethnic minority students. Contemporary School Psychology. 2017;21:223–39. doi: 10.1007/s40688-017-0134-1.

32. Crooks CV, Fortier A, Graham R, Hernandez ME, Chapnik E, Cadieux C, et al. Implementing a brief evidence-based tier 2 school mental health intervention: The enablers and barriers as seen through a clinical team supervisor lens. Canadian Journal of Community Mental Health. 2022;41:139–56. doi: 10.7870/cjcmh-2022-017.

33. Dowling K, Barry MM. Evaluating the implementation quality of a social and emotional learning program: A mixed methods approach. Int. J. Environ. Res. Public Health. 2020;17. doi: 10.3390/ijerph17093249.

34. Bailey SJ, Oosterhoff B, Lindow JC, Robecker T, Bryan B, Byerly MJ. Feasibility, acceptability, and fidelity: Extension agents teaching youth aware of mental health. Journal of Rural Mental Health. 2022;46:88–99. doi: 10.1037/rmh0000170.

35. O'Dea B, Subotic-Kerry M, King C, Mackinnon AJ, Achilles MR, Anderson M, et al. A cluster randomised controlled trial of a web-based youth mental health service in Australian schools. The Lancet Regional Health - Western Pacific. 2021;12. doi: 10.1016/j.lanwpc.2021.100178.

36. Chugani CD, Murphy CE, Talis J, Miller E, McAneny C, Condosta D, et al. Implementing Dialectical Behavior Therapy Skills Training for Emotional Problem Solving for Adolescents (DBT STEPS-A) in a Low-Income School. School Mental Health. 2022;14:391–401. doi:10.1007/s12310-021-09472-4.

37. Aguilar T, Espelage DL, Valido A, Woolweaver AB, Drescher A, Plyler V, et al. Lessons learned from implementing sources of strength: A qualitative examination of a peer-led suicide prevention program. School Mental Health: A Multidisciplinary Research and Practice Journal. 2023;15:812–25. doi: 10.1007/s12310-023-09587.

38. McAllister M, Withyman C, Knight BA. Facilitation as a vital skill in mental health promotion: findings from a mixed methods evaluation. Journal of Mental Health Training, Education & Practice. 2018;13:238–47. doi:10.1111/inm.12412.

39. Dariotis JK, Mabisi K, Jackson-Gordon R, Rose EJ, Fishbein DH, Mendelson T. Perceived Benefits of Mindfulness and Health Education Programs for Minoritized Adolescents: A Qualitative Analysis. Mindfulness. 2023;14:1346–61. doi: 10.1007/s11121-022-01481-2.
